# Ellagic Acid and Metformin: In Vitro Synergy and Enhanced Cisplatin Efficacy In Vivo in Resistant Breast Cancer

**DOI:** 10.3390/nu18183047

**Published:** 2026-09-17

**Authors:** Balqis Jdaitawi, Moudi M. Alasmari, Heba K. Alshaeri, Doaa Omar Anbarserry, Alexandra Makai, Hadeel Shaher Al Junaidi, Wamidh H. Talib, Márta Hock

**Affiliations:** 1 Department of Clinical Pharmacy and Therapeutics, Faculty of Pharmacy, Applied Science Private University, Amman 11937, Jordan; balqisjdaitawi0@gmail.com; 2 College of Medicine, King Saud bin Abdulaziz University for Health Sciences (KSAU-HS), Jeddah 11481, Saudi Arabia; asmarim@ksau-hs.edu.sa; 3 King Abdullah International Medical Research Centre (KAIMRC), Jeddah 11481, Saudi Arabia; 4 Department of Clinical Pharmacology, Faculty of Medicine, King Abdulaziz University, Rabigh 21589, Saudi Arabia; hkalshaeri85@gmail.com (H.K.A.); dserri@kau.edu.sa (D.O.A.); 5 Institute of Physiotherapy and Sports Science, Faculty of Health Sciences, University of Pécs, H-7621 Pécs, Hungary; alexandra.makai@etk.pte.hu (A.M.);; 6 Physical Activity Research Group, János Szentágothai Research Center, University of Pécs, H-7624 Pécs, Hungary; 7 Faculty of Allied Medical Sciences, Applied Science Private University, Amman 11937, Jordan

**Keywords:** breast cancer, metformin, ellagic acid, combination therapy, cisplatin resistance, apoptosis, combination index

## Abstract

Cisplatin resistance remains a significant challenge in the effective treatment of breast cancer, underscoring the need for novel combination-based therapeutic strategies. **Background**: This study investigated the anticancer effects of ellagic acid (EA) and metformin (MET), both individually and in combination, against parental and cisplatin-resistant breast cancer models. To our knowledge, this is the first study to evaluate the EA-MET combination in the context of cisplatin resistance in breast cancer. **Methods**: The in vitro antiproliferative and apoptotic effects of EA and MET were assessed using MTT and caspase-3 assays in parental (EMT-6/P) and cisplatin-resistant (EMT-6/CPR) cells. An in vivo study was also conducted to evaluate the anti-tumor effects of the treatment combinations in mice bearing EMT-6/P or EMT-6/CPR tumors. Liver enzymes and creatinine levels were measured to assess potential hepatic and renal toxicity. **Results**: In vitro combination index analysis indicated a synergistic interaction between EA and MET in EMT-6/P cells (CI = 0.45) and a strong synergistic interaction in EMT-6/CPR cells (CI = 0.29) under the tested conditions. In vivo, the addition of the combination to cisplatin resulted in significant tumor size reductions of 73.83% and 56.25% in EMT-6/P and EMT-6/CPR bearing mice, respectively, along with tumor regressions of 66.6% and 50%, respectively. **Conclusions**: Thecombination of metformin and ellagic acid shows promise as a potential treatment for cisplatin-resistant breast cancer. Further research is needed to elucidate the precise anticancer mechanisms underlying this combination.

## 1. Introduction

Cancer continues to be a predominant cause of morbidity and mortality on a global scale. In 2024, there were 20.6 million newly diagnosed cancer cases and 9.8 million deaths attributable to cancer [1]. A prominent type of cancer affecting women is breast cancer, which causes a high mortality rate worldwide and accounts for 6.9% of all cancer cases [2]. Without early detection and innovative treatment approaches to address emerging cases, experts predict that by 2050, more than 6 million new cancer cases will be diagnosed annually [3].

Chemotherapy still encounters major difficulties and frequently causes harmful side effects for patients [4]. Generally, the goal of these medications is to eradicate tumors and inhibit their proliferation to prevent growth and metastasis [5]. Among chemotherapy agents, platinum-based drugs such as cisplatin are used to treat multiple cancers, including head and neck, ovarian, bladder, testicular, prostate, lung, and breast cancers, and refractory non-Hodgkin’s lymphomas [6]. Although initially effective, drug resistance poses a significant barrier to successful cancer eradication and overall patient survival, as it leads to more aggressive tumor behavior and a poorer prognosis [7].

Cisplatin (CIS) resistance is common and often inevitable following a patient’s initial exposure to the drug, leading to treatment failure [8]. Therefore, it is essential to explore more sustainable treatment options, such as natural compounds, which are known for their diverse mechanisms to combat tumor progression while protecting normal cells from toxicity [9].

Ellagic acid (EA) is a natural polyphenolic compound found in various sources, including certain nuts, berries, and pomegranates [10]. It is widely recognized for its antineoplastic and antioxidative properties. For example, studies have demonstrated its efficacy in treating colon, lung, prostate, and breast cancers through mechanisms such as apoptosis induction, activation of the mitochondrial death pathway, and caspase-3 enzyme activation [11,12,13]. In addition to natural compounds, combination therapy using multiple agents has proven effective in treating resistant cancer types by targeting multiple pathways simultaneously, thereby reducing side effects and patient suffering [14].

Building on this approach, metformin (MET), an antidiabetic agent, has garnered interest due to increasing evidence of its antitumor effects. It acts against various tumors, including colorectal, lung, ovarian, prostate, and pancreatic cancers, by modulating critical cancer survival pathways such as mTOR, IGF, and AMPK. MET also influences immune activation, cellular apoptosis, and cancer metabolism [15,16]. Since Cisplatin-resistant breast cancer cells often show increased prosurvival signaling, metabolic reprogramming, and defective apoptotic responses, co-targeting these complementary pathways may re-sensitize the resistant cells to cisplatin and improve therapeutic efficacy [17]. Therefore, it is hypothesized that the combination of EA and MET will have synergistic antitumor effects by overcoming multiple resistance mechanisms that are unlikely to be effectively targeted by either agent alone. Therefore, this study was designed to evaluate the therapeutic potential and underlying mechanisms of this combination in both in vitro and in vivo models of CIS-sensitive and CIS-resistant breast cancer.

## 2. Materials and Methods

### 2.1. Instruments, Reagents, and Commercial Kits

Standard laboratory equipment was used in this study. Cell culture procedures were performed under sterile conditions in a laminar flow hood (Forma Scientific Incorporated, Marietta, OH, USA). Cells were cultured in a CO_2_ incubator (Lab-Line Instruments Incorporated, Melrose Park, IL, USA) and centrifuged using refrigerated and microcentrifuges. Cell viability and proliferation were assessed using a microplate reader (ELx800, BioTek, Shoreline, WA, USA). Metformin (MET, 99% purity) was obtained from Wanbury Ltd. (Navi Mumbai, India), and ellagic acid (EA) was obtained from Genochem-World (Bonrepòs i Mirambell, Spain). Cells were cultured in Minimum Essential Medium (MEM; Caisson, Colorado Springs, CO, USA) supplemented with L-glutamine (Eurobio, Les Ulis, France), MEM non-essential amino acids (Caisson, USA), fetal bovine serum (FBS; Sigma, Ronkonkoma, NY, USA), gentamicin (Sigma, USA), and penicillin-streptomycin solution (Eurobio, France). Adherent cells were detached with trypsin–EDTA (Eurobio, France), and washes were performed with phosphate-buffered saline (PBS; Eurobio, France).

Cell viability was assessed using the MTT assay (Sigma, USA), with trypan blue (0.4%; Sigma, USA) employed for cell counting and dimethyl sulfoxide (DMSO; Alpha Chemika, Maharashtra, India) used to dissolve the formazan crystals. Apoptosis was evaluated using a colorimetric caspase-3 assay kit (Sunlong Biotech, Hangzhou, China; catalog no. SL2079Hu). Serum creatinine, alanine aminotransferase (ALT), and aspartate aminotransferase (AST) levels were measured using commercially available kits (BioMajesty^®^, Holzheim, Germany). Buffered formalin (S.D. Fine-Chem Ltd., Mumbai, India) was used for tumor tissue preservation.

### 2.2. Cell Lines and Culture Conditions

From the European Collection of Cell Cultures (Salisbury, UK), we used two mouse mammary cell lines for this study: the cisplatin-resistant EMT-6/CPR and the parental mouse breast cancer cell line EMT-6/P. The cells were cultured in MEM supplemented with the substances mentioned above. Cell lines were maintained in a CO_2_ incubator at 37 °C, with 5% CO_2_ and 95% humidity, for the required time periods specified in the study [18].

### 2.3. Preparation of EA, MET, and CIS Working Solutions

The concentration ranges were chosen based on earlier in vitro studies that reported the antiproliferative effects of these compounds across various cancer models. These concentrations were selected to encompass the ranges described in the literature where biological activity had been observed, allowing for the assessment of concentration-dependent cytotoxic effects and the determination of IC_50_ values. Both EA and MET were selected based on prior studies demonstrating antiproliferative activity at 100 µM. EA had previously been tested in three metastatic melanoma cell lines (1205Lu, WM852c, and A375), showing inhibitory effects at concentrations ranging from 25 to 100 µM [19]. Similarly, MET was reported to significantly inhibit the progression of ovarian cancer cell lines at 100 µM [20]. Based on the MTT assay protocols, EA was prepared to achieve a final working concentration of 100 µM by first making a concentrated 66 mM stock solution in DMSO. Then, 3 µL of this stock was added to 970 µL of culture medium. For MET, the same final concentration of 100 µM was obtained by dissolving it directly in the culture medium. For single-agent treatments in both sensitive and resistant cells, successive 1:2 serial dilutions were performed to obtain concentrations of 100, 50, 25, 12.5, 6.24, 3.12, 1.56, and 0.078 µM. CIS, used as the reference treatment (Ebewe Pharma, Unterach am Attersee, Austria), was prepared as a 0.5 mg/mL stock solution. The concentration range chosen for CIS (from 10 µM down to 0.08 µM) was based on previous literature demonstrating that a 10 µM CIS dose caused the greatest reduction in cell viability in oral squamous cell carcinoma [21]. This concentration range was applied in individual treatment experiments for both resistant and normal breast cancer cells. Before use, the stock solution was diluted with MEM to achieve the target concentrations.

Combination dosing involved stepwise two-fold dilutions of EA or MET, with one compound held constant while the other was serially diluted. For CIS-resistant cells, EA ranged from 50 to 0.39 µM with MET fixed at 37.47 µM, and conversely, MET was diluted from 37.47 to 0.29 µM with EA held at 50 µM. For non-resistant BC cells, EA varied from 6.87 to 0.054 µM with MET fixed at 1.5 µM, and MET ranged from 1.5 to 0.05 µM with EA held at 6.87 µM [22].

### 2.4. Antiproliferative Assay (MTT)

Immediately after counting the cells, the cell suspension was prepared and seeded into 96-well tissue culture plates at a density of 15,000 cells/well in 100 µL of culture medium. Cells were incubated for 24 h under standard culture conditions (37 °C, 5% CO_2_, and 95% humidity) to allow cell attachment. Thereafter, the culture medium was replaced with fresh medium containing EA + MET + CIS or combinations of EA + MET at the concentrations described above. The treatment medium was removed, and 100 µL of fresh medium with MTT solution was added to each well. After 48 h of treatment, plates were incubated for 3 h to allow formazan crystals to form. The reaction was quenched with DMSO and incubated for 1 h to dissolve the crystals. Absorbance was measured on a microplate reader (BioTek, Winooski, VT, USA) at 550 nm. Cell viability was expressed as a percentage of the untreated control group, and IC_50_ values were calculated from concentration-response curves. All treatments were performed in technical triplicate within each experimental run, and the complete experiments were independently repeated three times. Thus, three independent experimental replicates were obtained for each assay. The three technical replicates within each independent experiment were averaged to generate a single value for that experiment, and the resulting three independent values were used for statistical analysis (*n *= 3).

### 2.5. IC_50_ Value Determination for Tested Compounds

IC_50_ is the concentration of a drug or compound required to reduce cell viability by 50% relative to an untreated control [23]. The cytotoxic activity of each compound against the EMT-6/P and EMT-6/CPR cell lines was evaluated using this parameter. In this study, IC_50_ values for individual drugs and combination regimens were analyzed by nonlinear regression analysis using SPSS (version 26; Chicago, IL, USA). Cell survival was determined by comparing treated cells to the untreated control group, which consisted of cells cultured in medium without treatment. Dose–response curves were plotted based on the percentage of cell survival, and IC_50_ values were defined as the concentrations that inhibited 50% of cell viability compared to the untreated control.

### 2.6. Combination Index (CI) Calculation

The triple combination (EA + MET + CIS) was not tested in vitro due to significant challenges in interpreting MTT assays, such as overlapping effects and increased matrix complexity, as well as the need for more rigorous modeling to adequately characterize drug interactions. Instead, this combination was evaluated in vivo for its antitumor efficacy relative to dual therapy within a complex biological model. This approach enabled the assessment of treatment effects in a living system, including systemic and biological interactions that cannot be fully replicated in a simple in vitro setting.

The following equation was used to determine the combination index (CI) of two agents, EA and MET, against the EMT-6/P and EMT-6/CPR cell lines, interpretations of CI values are shown in Table 1:

$$CI=1+\frac{\left(D\right)2}{\left(DX\right)2}+\frac{a\left(D\right)1\left(D\right)2}{\left(DX\right)1\;\left(DX\right)2}$$

where

(D_x_) 1 = EA IC_50_;
(D) 1 = (EA with MET) IC_50_;
(D_x_) 2 = MET IC_50_;
(D) 2 = (MET with EA) IC_50_;
“a = 0 for mutually exclusive or 1 for mutually nonexclusive interaction”.

Referring to previous studies, MET and EA each combat cancer in different ways. Consequently, we considered α to be 1. The following is an interpretation of CI results.

**Table 1:** Interpretation of combination index values [24].

CI Value	Interpretation
CI > 1.3	Antagonism
CI = 1.1–1.3	Moderate antagonism
CI = 0.9–1.1	Additive effect
CI = 0.8–0.9	Slight synergism
CI = 0.6–0.8	Moderate synergism
CI = 0.4–0.6	Synergism
CI = 0.2–0.4	Strong synergism
CI < 0.1	Very strong synergism

The resistance fold is used to compare resistant and parental cell lines; it is the concentration needed to kill 50% of the resistant cell line relative to the parental cell line. In this work, the resistance fold is determined by the following formula to compare the IC_50_ values of resistant cell lines with those of sensitive parental BC cell lines [25]:

$$Resistance\;fold=\frac{IC50\;of\;resistant\;cell\;line}{IC50\;of\;parental\;cell\;line\;}$$

### 2.7. Apoptotic Activity in EMT-6/P and EMT-6/CPR Cells as Determined by Colorimetric Assay

The activity of caspase-3 was detected using a human CASP-3 ELISA kit (Sunlong Biotech, Cat. No. SL2079Hu) according to the manufacturer’s instructions. EMT-6/CPR cells were cultured in standard conditions (37 °C, 5% CO_2_, 95% humidity) and treated for 48 h with EA (13.74 µM), MET (3.06 µM), CIS (3.81 µM), and the EA + MET combination. Untreated cells served as controls. The cells were collected post-treatment, lysed by repeated freeze–thaw cycles, and centrifuged to obtain protein extracts. The levels of caspase-3 were measured by ELISA. The assay was independently repeated in three separate experimental runs, with each experimental run performed in technical triplicate. Caspase-3 activity was calculated as relative fold changes compared to the untreated control group. The fold increase after each treatment was calculated using this formula.

$$Fold\;increase=\frac{caspase\;concentration\;(treated)}{caspase\;concentration\;(control)}$$

### 2.8. Experimental Animals

For this study, 66 female BALB/c strain mice, each weighing 21–25 g and aged 4–6 weeks, were used because the EMT-6/P and CPR breast cancer cell lines were originally derived from this strain, making them syngeneic. The mice were supplied by the Applied Science Private University’s animal house in Amman, Jordan. All animal experimental procedures were approved by the Applied Science University Research and Ethics Committee in compliance with recognized ethical guidelines (2025-PHA-19). The animals were housed in separate cages with wood shavings as bedding. The animal housing was maintained at a constant temperature of 25 degrees Celsius, with humidity between 50 and 60 percent, continuous air circulation, and 12-h light/dark cycles.

### 2.9. Setting Up Procedures for the Animal Experiment

The in vivo study on female BALB/c mice bearing parental and cisplatin-resistant breast cancer (BC) tumors was conducted. Treatment dosages were selected based on a thorough review of the literature. The EA concentration was 45 mg/kg/day, as reported in references [26,27]. MET was administered at 80 mg/kg/day, according to reference [28], and CIS was given at 0.7 mg/kg/day, based on reference [25].

### 2.10. Tumor Cell Implantation and In Vivo Anticancer Activity Testing

After being thawed, both parental breast cancer (BC) cells and CIS-resistant cells were cultured separately and quantified. Using appropriate media, the cells were then prepared for inoculation. Thirty mice were assigned to the parental (non-resistant) tumor model, and thirty-six mice were assigned to the resistant tumor model. Each animal received a 0.1 mL of subcutaneous injection containing 100,000 cells. The 36 mice with the resistant breast cancer cell line were injected with an equivalent volume of EMT-6/CPR cell suspension. To allow tumor formation, all mice were maintained under standard conditions for 14 days. Tumor diameters were subsequently measured using a digital caliper, and volumes were calculated using the formula below [29]:

Volume=A∗B∗B∗0.5

Here, A is defined as the tumor’s maximum length, while B is the measurement taken perpendicular to A.

In the mice injected with EMT-6/P, mice with similar tumor volumes were randomly allocated into five groups for EMT6/P and 6 groups for EMT6/CPR (*n* = 6 per group, and a total of 66 mice) to ensure comparable baseline tumor burdens across treatment groups. The treatment allocation was not blinded, and we were aware of the treatment assigned to each group during tumor measurement and outcome assessment. The following intraperitoneal (IP) injections were part of the therapy regimen, which started on day 15 and lasted for a week (Table 2):

▪ Group 1 was injected with EA dissolved in 0.1% DMSO in PBS.
▪ Group 2 was injected with MET dissolved in the media.
▪ Group 3 was injected with a combination of EA and MET.
▪ Group 4 was injected with a combination of EA, MET, and CIS.
▪ Group 5 was the control group, injected with media without any drug.

In the mice injected with EMT-6/CPR, those with similar tumor sizes were allocated into six groups of six animals each. The following intraperitoneal (IP) injections were part of the therapy regimen, which started on day 15 and lasted for a week (Table 2):

▪ Group 1 was injected with EA dissolved in 0.1% DMSO in PBS.
▪ Group 2 was injected with MET dissolved in the media.
▪ Group 3 was injected with CIS only.
▪ Group 4 was injected with a combination of EA and MET.
▪ Group 5 was injected with a combination of EA, MET, and CIS.
▪ Group 6 was the control group, injected with media without any drug.

Three blood samples were collected on days 1, 5, and 10 during the 10-day treatment course.

Tumor volumes were measured three times during the treatment period (on days 1, 5, and 10). The percentage change in tumor volume between the initial and final measurements was calculated using the formula below [30]:

$$\%\;Tumor\;change=\left(\frac{F-1}{1}\right)*100\%$$

where F denotes the final tumor volume, and 1 denotesthe initial tumor volume.

Blood samples were obtained retro-orbitally from each group of mice ten days after treatment, following euthanasia by cervical dislocation. To preserve their morphology, the tumors were fixed in 10% formalin. For blood sample preparation, the samples were centrifuged at 5000 rpm for 10 min, and the resulting serum was transferred into freshly labeled Eppendorf tubes. The collected serum samples were stored at −20 °C until further analysis.

### 2.11. Assessment of Hepatic and Renal Function in Treated Mice

To determine the potential hepatic and renal effects of EA, MET, CIS, and their combinations, serum levels of alanine aminotransferase (ALT), aspartate aminotransferase (AST), and creatinine were measured in all treatment groups and the negative control group using commercial assay kits, following the manufacturers’ instructions. Working reagents were freshly prepared and pre-incubated at 37 °C before analysis. For the ALT and AST assays, 100 µL of serum was mixed with 1 mL of working reagent, and absorbance was measured at 340 nm at predetermined time points. Serum creatinine was determined by adding 100 µL of serum to 1 mL of working reagent, with absorbance measured at 505 nm. For all assays, distilled water was used as the blank reference to calibrate the spectrophotometer before measurement [31].

### 2.12. Statistical Analysis

SEM (Standard Error of the Mean) was used to represent the data. After treating parental and CIS-resistant cell lines, EA, MET, CIS, and their combinations were evaluated to determine their IC_50_ values. Additionally, the IC_50_ values were analyzed using nonlinear regression of dose–response curves in SPSS (Statistical Package for the Social Sciences, Chicago, IL, USA). Normality of the data was evaluated using the Shapiro–Wilk test, and homogeneity of variances was evaluated using Levene’s test. The assumptions for parametric analysis were met, and one-way ANOVA, followed by Dunnett’s post hoc test for comparisons between each treatment group and the control, and Tukey’s post hoc test for comparisons between the triple-combination group and the other treatment groups, was used to assess differences among treatment groups. Statistical significance was considered at a *p*-value < 0.05. Methods were performed as previously described by Hamed et al., with minor modifications [25]. All in vitro experiments were conducted in technical triplicate for each experimental run, and the whole experiments were conducted independently three times. Thus, each assay was performed in three independent experimental replicates. For each independent experiment, the three technical replicates were averaged to give one value for that experiment, and the three independent values obtained were used for statistical analysis (*n* = 3).

## 3. Results

### 3.1. In Vitro Findings

Cisplatin-sensitive and cisplatin-resistant breast cancers were modeled in vitro using the murine mammary carcinoma cell lines EMT-6/P and EMT-6/CPR, respectively. Because these cell lines share a similar genetic background, they provide a reliable system to evaluate treatment effects associated with cisplatin resistance. Additionally, EMT-6/P cells were used in the in vivo study. Together, these models offer a biologically relevant framework for assessing the anticancer efficacy of MET and EA, both individually and in combination, against cisplatin-resistant and cisplatin-sensitive breast cancer.

#### 3.1.1. EA and MET Reduced the Viability of EMT-6/P and EMT-6/CPR Cells

**Figure 1:**
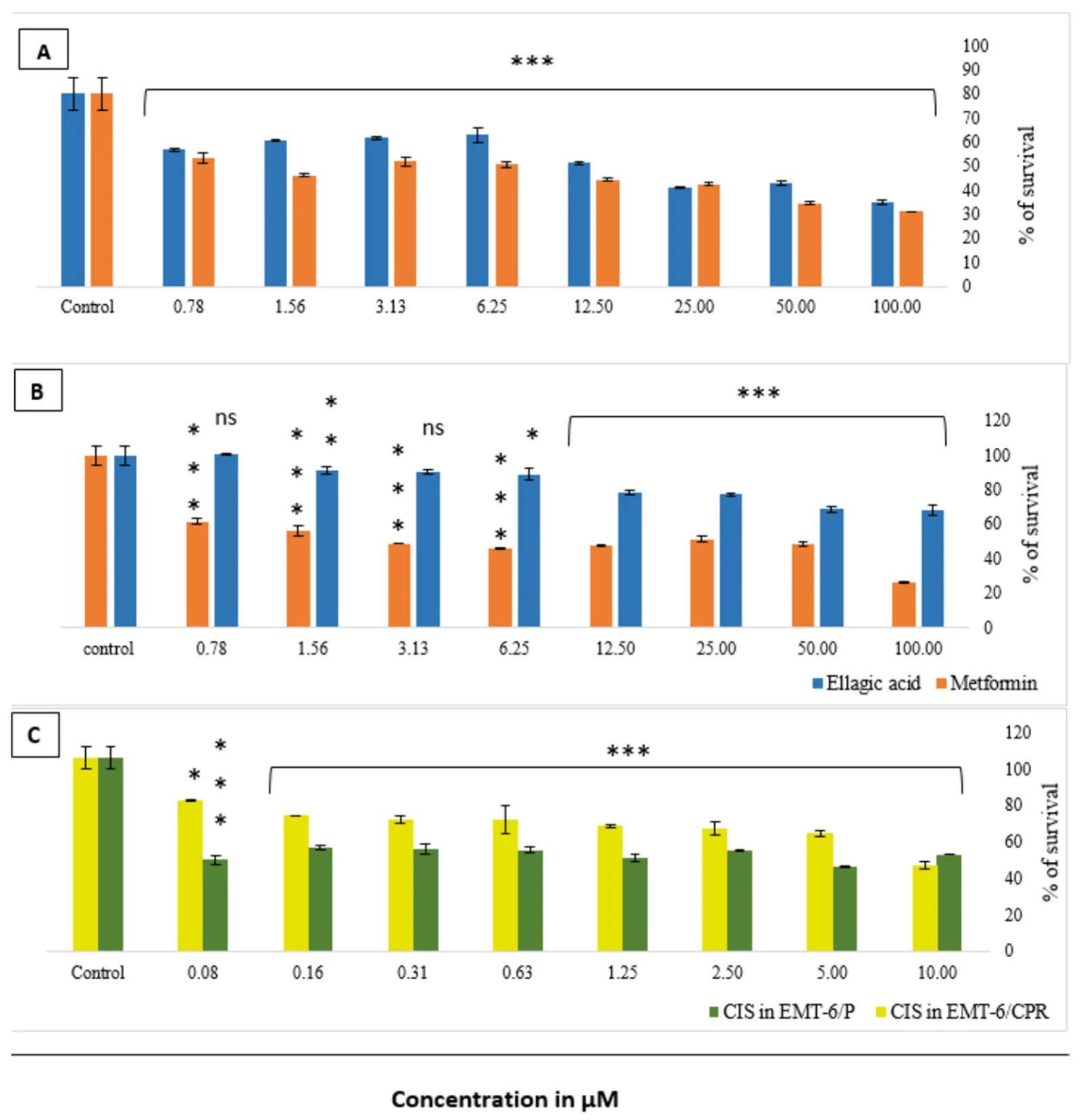
Antiproliferative effects of individual treatments on EMT-6/P and EMT-6/CPR cells. (**A**) Individual treatment of EMT-6/P cells with ellagic acid (EA) and metformin (MET). (**B**) Individual treatment of EMT-6/CPR cells with EA and MET. (**C**) Effect of cisplatin (CIS) treatment on EMT-6/P and EMT-6/CPR cells. Data are expressed as mean ± SEM from three independent experiments, each performed in technical replicates (*n* = 3). Statistical significance was determined by comparing each concentration with the untreated control within the corresponding cell line. Significant differences are indicated as * *p* < 0.05, ** *p* < 0.01, and *** *p* < 0.001 versus control.

Both cell lines were treated with increasing concentrations of EA and MET (0.78–100 µM). EA and MET reduced viability in EMT-6/P cells in a concentration-dependent manner; however, MET exhibited greater cytotoxic activity than EA. The IC_50_ values of EA and MET in EMT-6/P cells were 13.74 ± 0.89 µM and 3.06 ± 3.38 µM, respectively (Figure 1A). In EMT-6/CPR cells, both agents reduced cell viability; however, cell survival was significantly higher following EA treatment at all tested concentrations. MET exhibited moderate inhibitory activity, with an IC_50_ value of 74.93 ± 11.03 µM, whereas the IC_50_ value for EA exceeded 100 µM, indicating lower sensitivity of EMT-6/CPR cells to EA treatment (Figure 1B).

#### 3.1.2. EMT-6/CPR Cells Exhibited Increased Resistance to Cisplatin Compared to EMT-6/P Cells

CIS was applied to both cell lines at concentrations of 10, 5, 2.5, 1.25, 0.63, 0.31, 0.16, and 0.08 µM for 48 h. The anti-proliferative activity of CIS on these cell lines was evaluated using the MTT assay. The IC_50_ was determined to be 3.81 ± 0.7 µM for the EMT-6/P cell line and 9.68 ± 2.5 µM for the EMT-6/CPR cell line. Based on these values, the resistance fold of EMT-6/CPR cells was 2.54-fold, which indicates that EMT-6/CPR cells were more resistant to CIS than EMT-6/P cells, as shown in Figure 1C and Table 3.

In the EA treatment model, EMT-6/CPR cells exhibited 7.27-fold higher resistance than EMT-6/P cells. MET exhibited the highest resistance fold, with EMT-6/CPR cells demonstrating a 24.48-fold increase in resistance relative to the parental EMT-6/P cell line.

#### 3.1.3. Co-Treatment with EA and MET Suppressed Proliferation in Both EMT-6/P and EMT-6/CPR Cells

**Figure 2:**
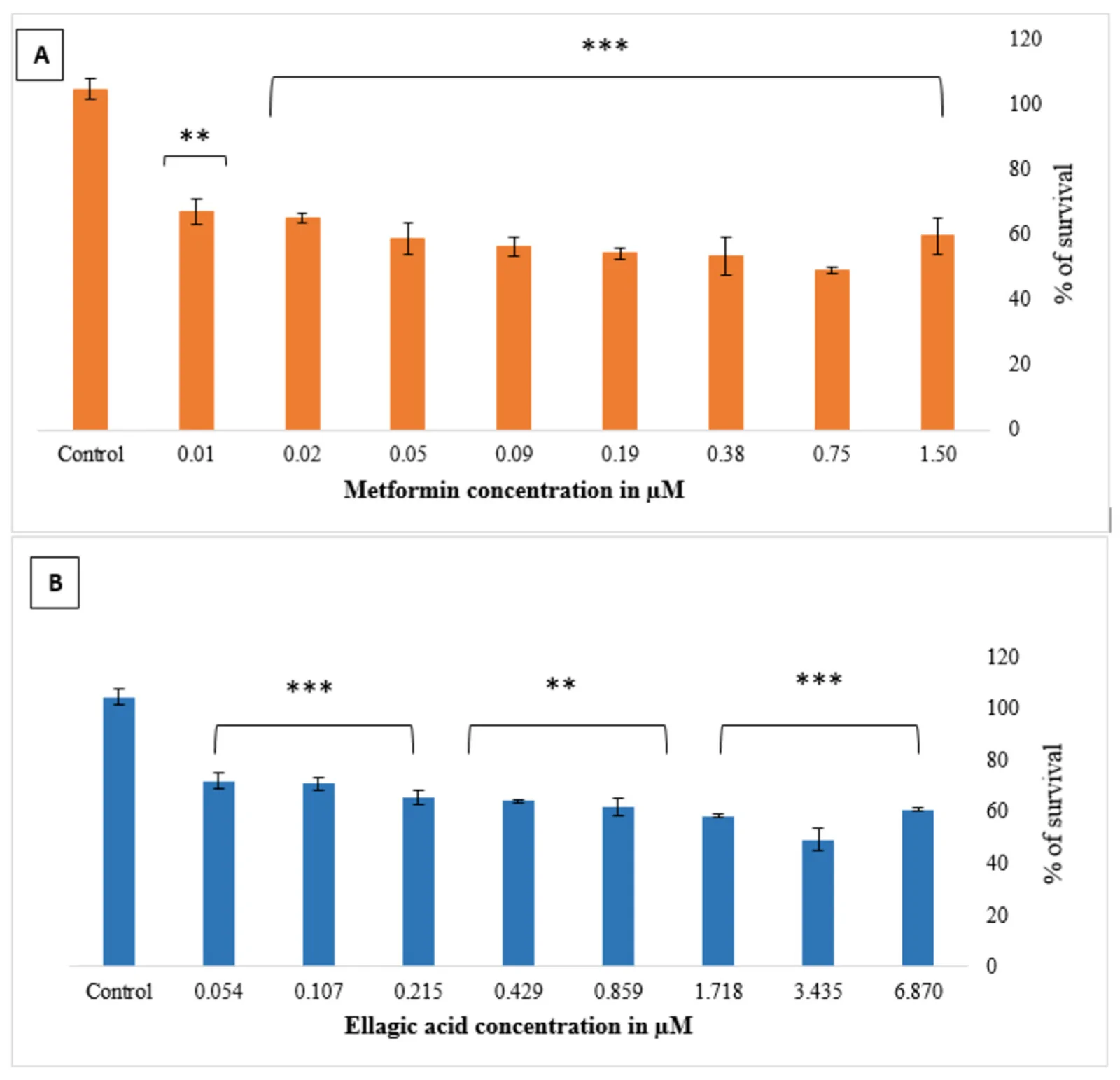
Antiproliferative effects of combination treatments on EMT-6/P cells. (**A**) Combination treatment of MET with 6.87 µM EA. (**B**) Combination treatment of EA with 1.5 µM MET. Each concentration was compared to the negative control. Data are presented as mean ± SEM from three independent experiments, each performed in technical replicates (*n* = 3). Error bars represent SEM. Significant differences are indicated as ** *p* < 0.01, *** *p* < 0.001, versus control.

**Figure 3:**
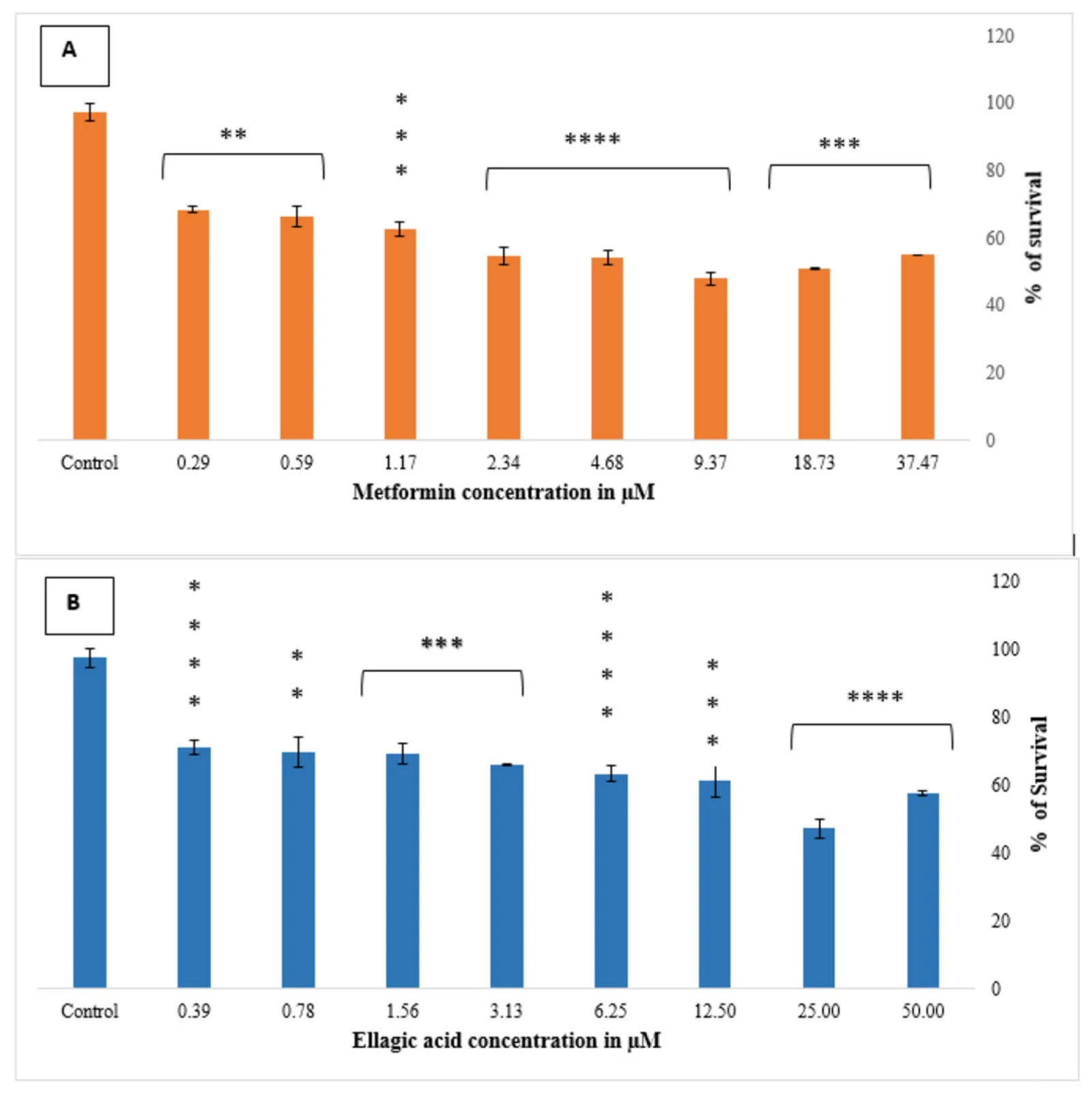
Antiproliferative effects of combination treatments on EMT-6/CPR cells. (**A**) Combination treatment of MET with 50 µM EA. (**B**) Combination treatment of EA with 37.47 µM MET. Each concentration was compared to the negative control. Data are presented as mean ± SEM from three independent experiments, each performed in technical replicates (*n* = 3). Error bars represent SEM. Significant differences are indicated as ** *p* < 0.01, *** *p* < 0.001, and **** *p* < 0.0001 versus control.

Parental EMT-6/P cells were treated with varying concentrations of EA while maintaining a fixed dose of MET, and vice versa, for 48 h to evaluate the inhibitory effects of combining EA and MET. The combination of EA and MET decreased cell viability, as demonstrated by the MTT assay results (Figure 2). This combination notably reduced the concentration required to reach the IC_50_ compared to individual treatments. The combinations with the lowest IC_50_ values involved serial dilutions of MET. EMT-6/P cells had IC_50_ values of 3.57 ± 0.89 µM for EA and 0.52 ± 0.23 µM for MET, whereas EMT-6/CPR cells had IC_50_ values of 16.31 ± 4.69 µM for EA and 8.34 ± 2.1 µM for MET (Figure 3).

#### 3.1.4. Combination of EA and MET Attenuated Resistance and Showed Synergistic Effects in EMT-6/CPR Cells

CI was quantified using the previously mentioned equation. CI values were 0.45 and 0.25, respectively, for the parental and CIS-resistant BC cells. The CI indicated a synergistic interaction in EMT6/P and a strong synergistic interaction in EMT6/CPR cells.

To assess the level of resistance in EMT-6/CPR cells, the MTT assay was used to compare the IC_50_ values of EA, MET, and CIS in both cell lines, individually and in combination. EMT-6/CPR cells exhibited approximately 7.2-fold higher resistance to EA than EMT-6/P cells, as demonstrated by the IC_50_ values of 13.74 µM in EMT-6/P cells and 100 µM in EMT-6/CPR cells. Regarding MET, EMT-6/CPR cells showed 24.48-fold higher resistance, with IC_50_ values of 3.06 µM for EMT-6/P and 74.93 µM for EMT-6/CPR cells.

In contrast, the IC_50_ values for CIS were 3.81 µM and 9.68 µM for EMT-6/P and EMT-6/CPR cells, respectively. This indicates that EMT-6/CPR cells were 2.54 times more resistant to cisplatin compared with EMT-6/P cells. For the combination treatment, the resistance fold for EA was reduced to 4.56, and for MET, it decreased to 16.03. The resistance fold was calculated by dividing each treatment’s IC_50_ in the resistant EMT-6/CPR cell line by its IC_50_ in the sensitive EMT-6/P cell line, as shown in Table 3.

#### 3.1.5. EA, MET, CIS, and Their Combination Modulated Caspase-3 Activity in EMT-6/P and EMT-6/CPR Breast Cancer Cells

**Figure 4:**
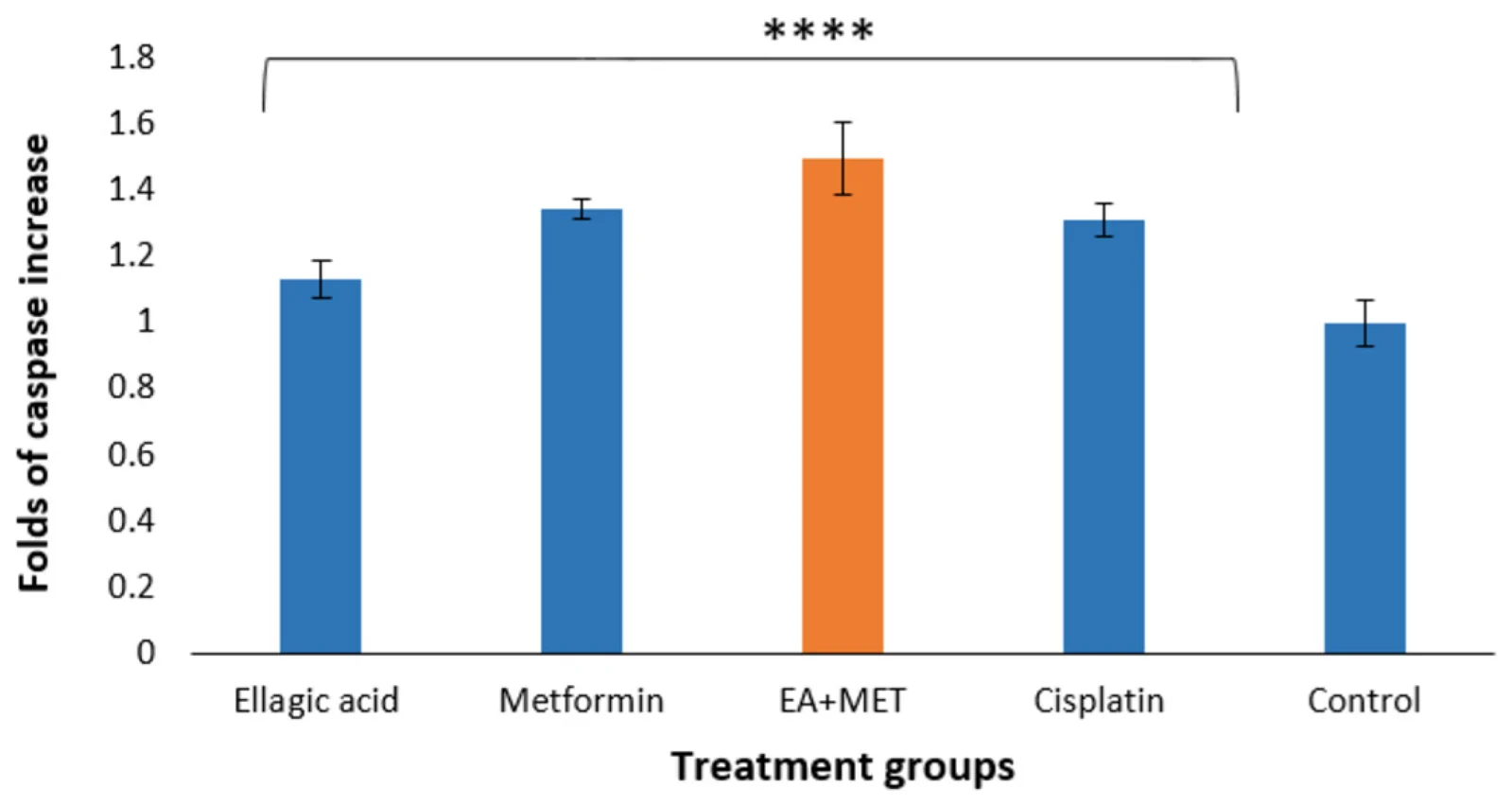
Caspase-3 activity in EMT-6/CPR cells following treatment with ellagic acid (EA), metformin (MET), cisplatin (CIS), and combination therapy (EA + MET). Caspase-3 activity is expressed as a fold change relative to the untreated control. Data are presented as mean ± SEM from three independent experiments, each performed in technical replicates (*n* = 3). Significant difference is indicated as **** *p* < 0.0001 versus control.

The apoptotic effects of EA, MET, CIS, and their combinations on caspase-3 activity in the EMT-6/CPR cell line were evaluated using a colorimetric caspase-3 assay kit. Caspase-3 activity showed a significant increase in all treatment groups compared to the control. The combination of EA and MET produced the most pronounced increase in caspase-3 activity, followed by CIS, MET, and EA alone, respectively. The EA and MET combination induced the largest increase in caspase-3 activity (1.5-fold). The MET and CIS groups exhibited similar fold increases (1.347 and 1.312, respectively), while the EA group showed a moderate elevation (1.132-fold). The control group remained at baseline (1-fold), as illustrated in Figure 4, where *p* < 0.0001.

**Figure 5:**
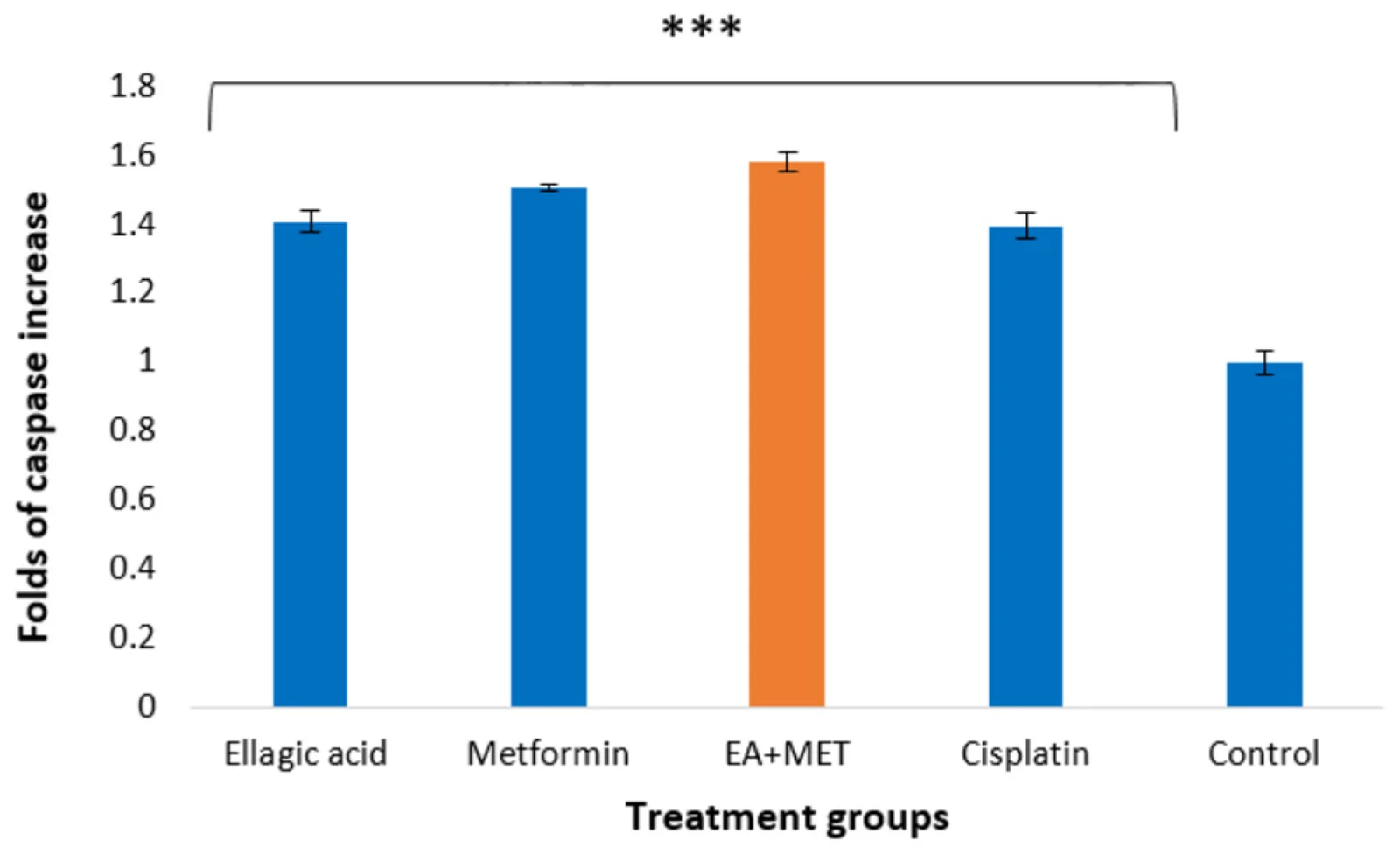
Caspase-3 activity in EMT-6/P cells following treatment with ellagic acid (EA), metformin (MET), cisplatin (CIS), and combination therapy (EA + MET). Caspase-3 activity is expressed as fold change relative to the untreated control. Data are presented as mean ± SEM from three independent experiments, each performed in technical replicates (*n* = 3). Significant difference is indicated as, *** *p* < 0.001 versus control.

The EMT-6/P cells exhibited similar patterns. The combined treatment increased caspase-3 activity by 1.581-fold, while EA and MET individually increased activity by 1.505-fold and 1.409-fold, respectively. The control remained at one-fold, and CIS also caused an increase to 1.395-fold, as shown in Figure 5, where the *p*-value was less than 0.001.

### 3.2. In Vivo Results

#### 3.2.1. Antitumor Effects of EA, MET, CIS, and Their Combination Treatments in EMT-6/CPR and EMT-6/P-Implanted Mice

EA, MET, and their combination were evaluated in an in vivo trial to investigate their potential therapeutic effects against breast cancer, following promising in vitro results. Both sensitive and resistant cell lines were inoculated into female Balb/c mice to establish a more physiologically relevant model. We assessed the effects of each treatment by monitoring tumor growth dynamics throughout the treatment period. The following results demonstrate the therapeutic impact of each agent on EMT-6/CPR and EMT-6/P tumors.

The control group in the EMT-6/CPR tumor-bearing mice exhibited a significant increase in tumor volume, indicating aggressive tumor growth in the absence of treatment. This group showed the highest average tumor weight (0.529 g), an average percent increase of 109.04%, and a final tumor size of 721.68 mm^3^.

Treatment with EA, MET, CIS, and their combinations produced varying effects on tumor progression. Mice treated with EA alone had an average tumor weight of 0.195 g, accompanied by a slight decrease of 2.7% in tumor volume. At the end of the trial, 33.3% of the mice were tumor-free. MET monotherapy resulted in a more significant reduction in tumor volume (28.34%) and decreased tumor weight to 0.298 g, with 16.6% of the animals being tumor-free.

CIS monotherapy resulted in a 17.7% reduction in tumor volume and decreased tumor weight to 0.43 g.

**Figure 6:**
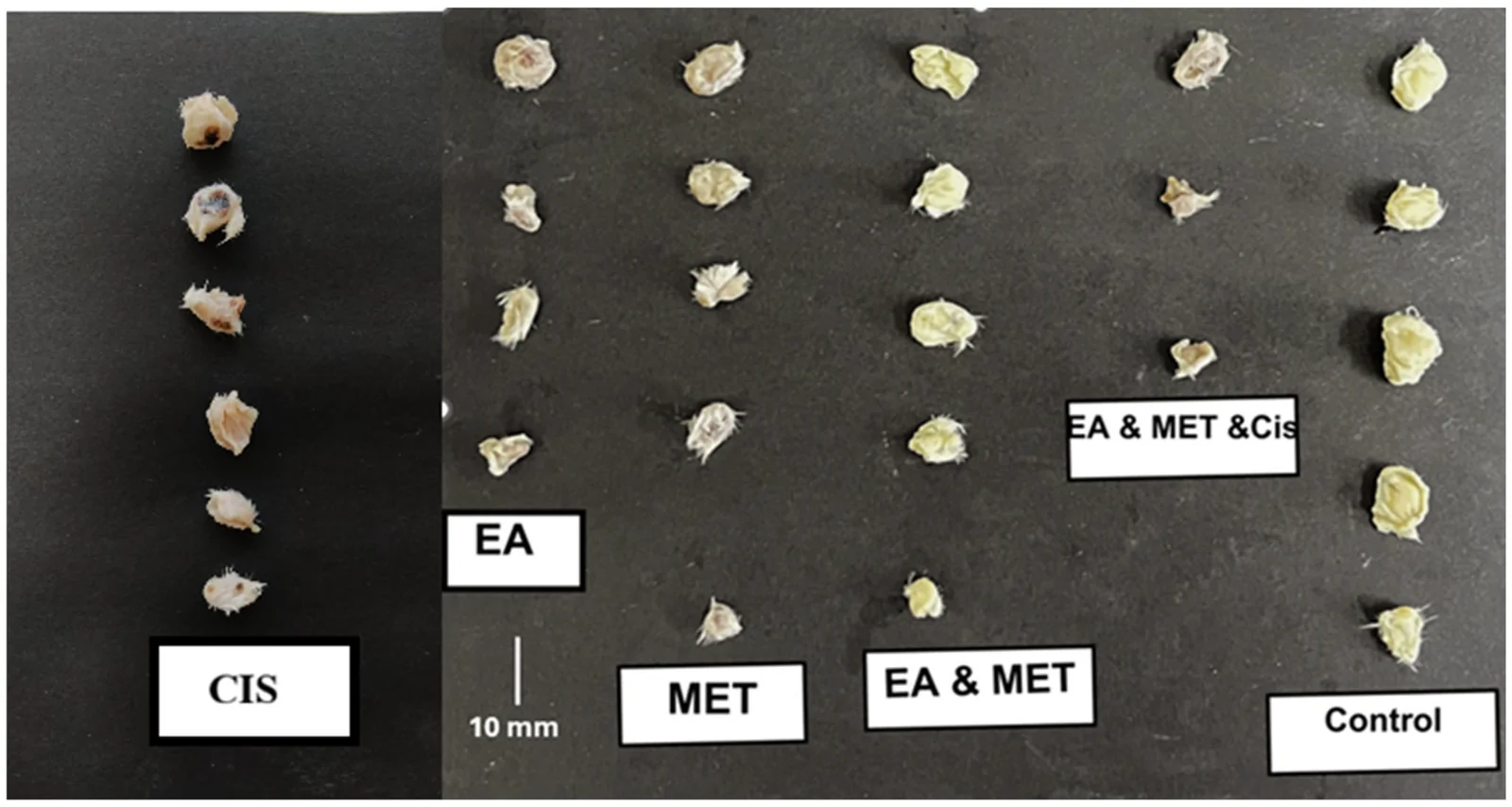
Tumor volumes in EMT-6/CPR mice were measured on day 10 post-dissection (*n* = 6 per group). In the EA + MET + CIS group, three tumors were undetectable, while two tumors were undetectable in the EA group. One tumor in each group was undetectable: MET, EA + MET, and control. Scale bar ≈ 10 mm.

**Figure 7:**
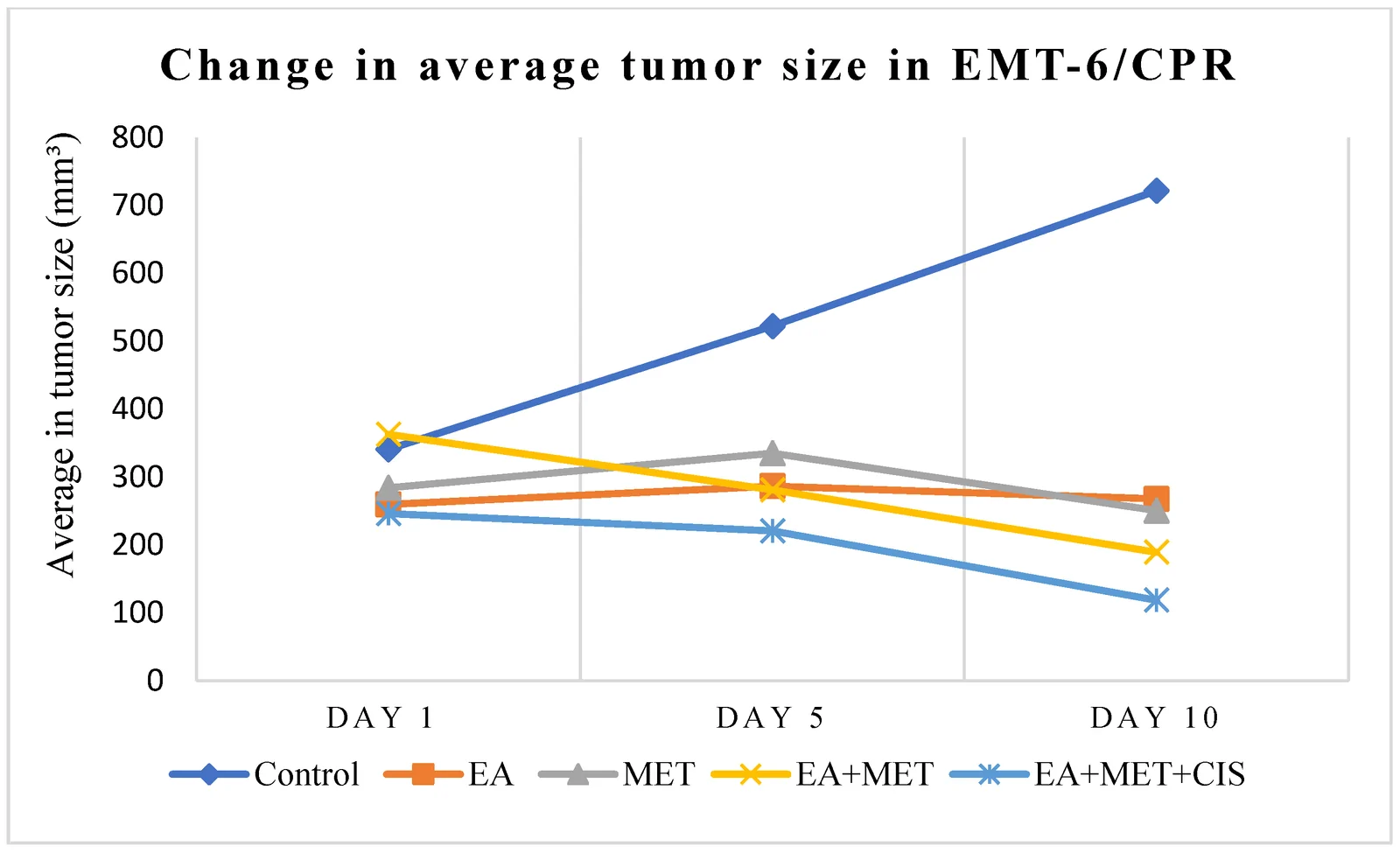
Average tumor size (mm^3^) vs. time (days) of treatment in the EMT-6/CPR cell line.

The anticancer effect was enhanced by the combination of EA and MET, as evidenced by a smaller tumor weight (0.171 g) and a 35.2% reduction in tumor volume compared to MET alone. Both the EA + MET + CIS combination and the MET monotherapy groups had the same proportion of tumor-free mice (16.6%), as shown in Table 4 and Figure 6 and Figure 7.

**Figure 8:**
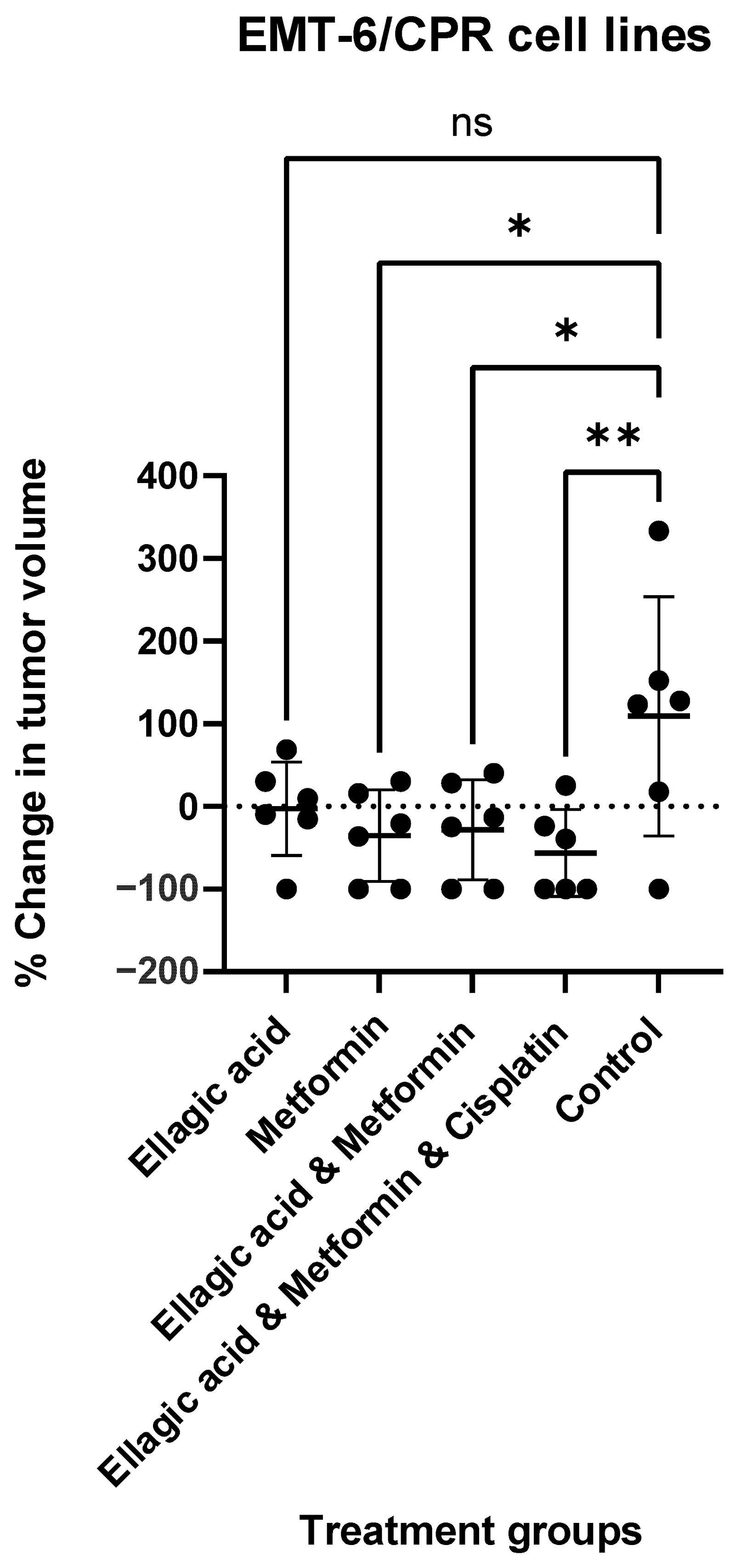
Percentage change in tumor volume in the EMT-6/CPR cell line after treatment with EA, MET, EA + MET, EA + MET + CIS. Data are presented as mean ± SEM from three independent experiments, each performed in technical replicates (*n* = 3). Significant differences are indicated as * *p* < 0.05, ** *p* < 0.01, and ns (not significant). This graph was generated using GraphPad Prism.

Figure 8 represents the percentage change in tumor volume in EMT-6/CPR tumor-bearing mice after treatment with EA, MET, and their combinations with or without CIS. Statistical analysis revealed that all treatment groups had significant reductions in tumor volume compared to the control group, except for the EA monotherapy group (ns). The combination treatment groups showed the most potent antitumor effects, with the strongest statistical significance for EA + MET + CIS (*p* < 0.01) and significantly reduced tumor volume for MET alone and EA + MET (*p* < 0.05). Data analysis and graphical work were performed using GraphPad Prism 9.5.1.

The antitumor activity of the combination of EA + MET + CIS was significantly greater than that of the MET and CIS monotherapy groups (*p* < 0.05). It was also significantly higher than that of the EA and CIS monotherapy groups (*p* < 0.0005). However, no significant difference was observed between the EA + MET + CIS combination and the EA monotherapy group (*p* > 0.05), indicating that the addition of MET and CIS did not result in a statistically significant improvement over EA alone under the tested conditions.

In the EMT-6/P cell line, the highest antitumor efficacy was observed in the (EA + MET + CIS) group, which exhibited a significant decrease in average tumor weight (0.088 g) and a tumor volume reduction of 56.25%. Additionally, this group had the highest percentage of tumor-free mice (50%).

**Figure 9:**
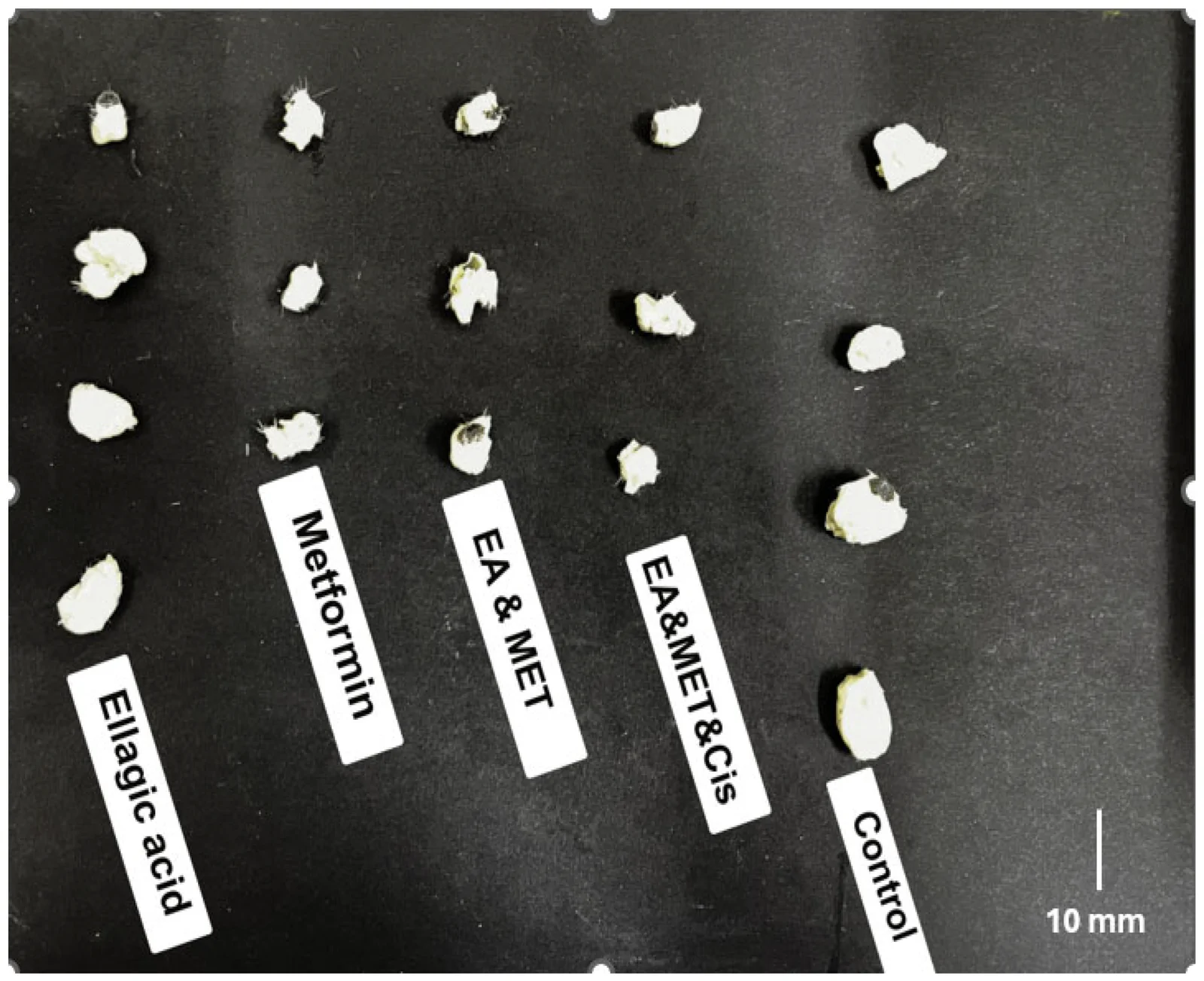
Tumor volumes in EMT-6/P mice were measured on day 10 post-dissection (*n* = 6 per group). Three tumors were undetectable in the MET, EA + MET, and EA + MET + CIS groups, while two tumors were undetectable in the EA and control groups. Scale bar ≈ 10 mm.

**Figure 10:**
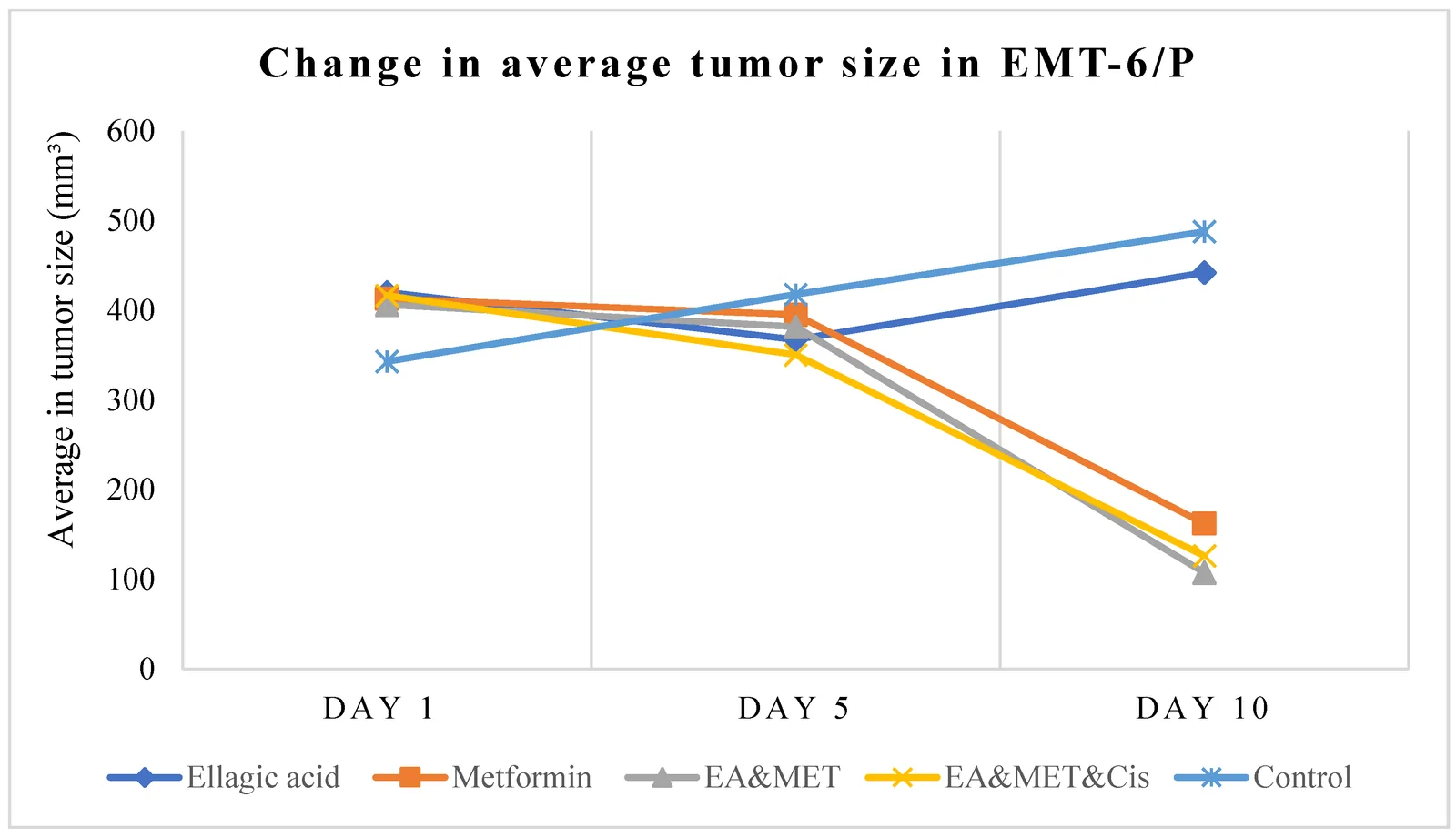
Average tumor size (mm^3^) vs. time (days) of treatment in EMT-6/P cell line.

When agents were applied to EMT-6/P tumors, the combination of EA + MET + CIS resulted in the greatest reduction in tumor volume (−73.83%), followed by the group receiving EA plus MET (−68.98%) and MET alone (−67.58%). These reductions corresponded with significantly lower average tumor weights (0.173 g, 0.165 g, and 0.150 g, respectively) and higher percentages of mice with no detectable tumors (66.6% in both combination groups and 50% in the MET group). EA alone produced the largest average tumor weight (0.429 g) and the smallest reduction in tumor size (−0.18%). In contrast, the control group exhibited the greatest increase in tumor size (+25.07%) and the highest average tumor weight (0.537 g) (Figure 9 and Figure 10). MET monotherapy exhibited significant antitumor activity, even greater than that of CIS, as indicated by the noted reductions in tumor volume and tumor weight compared with the control group. Combination treatments also showed strong inhibition, but effects on tumor reduction were not significantly greater than those of MET alone. The EA + MET + CIS group showed a significantly greater reduction in tumor burden than the EA monotherapy group (*p* < 0.0001), the MET monotherapy group (*p* < 0.05), and the untreated control group (*p* < 0.0001). However, no significant difference in tumor size was observed between the EA + MET + CIS group and the EA + MET group (*p* > 0.05), indicating that the addition of cisplatin to EA + MET did not result in a statistically significant further decrease in tumor size under the tested conditions, as demonstrated in Table 5.

**Table 5:** Tumor Size, Percent Change, and Average Weight in EMT-6/P Cells Following EA, MET, Combination, and CIS Treatments.

Treatment Group EMT-6/P	Av. Initial Tumor Size (mm^3^)	Av. Final Tumor Size (mm^3^)	%Change in Tumor Size (%)	Mice with No Detectable Tumor (%)	Av. Tumor Weight (g)	*p*-Values (EA + MET + CIS vs. Each Treatment)
EA	365.88	421.76	−0.18	16.6	0.429	<0.0001
MET	413.09	162.42	−67.58	50	0.150	<0.05
EA + MET	406.35	107.61	−68.98	66.6	0.165	ns
EA + MET + CIS	416.8	126.4	−73.835	66.6	0.173	-
Control	342.98	487.86	+25.07	33.3	0.537	<0.0001

Abbreviations: Av. = average; mm^3^ = cubic millimeter.

#### 3.2.2. Evaluation of Liver and Kidney Function in Mice

Serum AST and ALT levels were measured to assess potential hepatotoxicity caused by different treatments in EMT-6/CPR tumor-bearing mice. The mean AST value in the control group was 156.8 ± 7.57 IU/L. AST levels in the EA (201.47 ± 1.34 IU/L) and MET (193.9 ± 3.3 IU/L) treatment groups were significantly higher than those in the control group (*p* = 0.001 and *p* = 0.005, respectively). The EA + MET combination (150.1 ± 10.4 IU/L) did not differ significantly from the control group (*p* = 0.956). The highest AST level (234 ± 6.08 IU/L) was observed in the triple-combination group (EA + MET + CIS), which was significantly higher than that in the control group (*p* < 0.0001). Moreover, AST in the triple-combination group was significantly higher than that in the EA (*p* = 0.035), MET (*p* = 0.009), and EA + MET (*p* < 0.0001) groups.

A similar pattern was observed for ALT. The mean ALT level of the control group was 29.3 ± 1.92 IU/L. EA treatment (34.1 ± 4.2 IU/L) did not result in a significant difference when compared to the control group (*p* = 0.705), whereas MET (45.97 ± 1.4 IU/L) and the triple combination (50.3 ± 3.2 IU/L) resulted in significantly higher ALT levels when compared to the control group (*p* = 0.004 and *p* = 0.001, respectively). There was also no significant difference between the EA + MET combination (28.9 ± 4.56 IU/L) and the control group (*p* = 0.450). ALT levels in the triple-combination group were significantly higher than those in the EA group (*p* = 0.004) and the EA + MET group (*p* = 0.002), whereas the difference between the triple-combination group and the MET group was not significant (*p* = 0.751).

The mean creatinine level in the control group was 0.147 ± 0.01 mg/dL. The values were 0.15 ± 0.02 mg/dL in the EA group, 0.158 ± 0.01 mg/dL in the MET group, 0.15 ± 0.02 mg/dL in the EA + MET group, and 0.187 ± 0.001 mg/dL in the triple-combination group. There were no significant differences in the creatinine levels between any of the treatment groups when compared with the control group (EA, *p* = 0.743; MET, *p* = 0.537; EA + MET, *p* = 0.800; triple-combination, *p* = 0.271. Moreover, the triple-combination treatment did not differ significantly from EA (*p* = 0.779), MET (*p* = 0.962), or EA + MET (*p* = 0.691) (Table 6) [4,32].

## 4. Discussion

Although chemotherapy remains a critical cornerstone in cancer treatment, the challenges of chemoresistance and lack of selectivity create an urgent need to develop new strategies to overcome these obstacles [33]. While many tumors initially respond to chemotherapy and shrink during early treatment phases, the development of CIS resistance often becomes inevitable after multiple doses. Furthermore, CIS is associated with significant toxicities and side effects, making treatment tolerance difficult [34]. This provides the theoretical basis for combination therapies, which aim to reduce the required dosage, sensitize tumors to the agents, improve tolerability, and enhance anticancer efficacy [35].

It has been demonstrated that MET, when combined with CIS, acts as a potent chemo-sensitizing agent for various solid tumors, including breast cancer [36]. Regarding EA, studies have shown its ability to prevent CIS resistance in the ovarian cancer cell line A2780 [37]. In this study, we investigated the combined potential of these two compounds to overcome CIS resistance, a combination that has not been previously tested. The compounds were evaluated in vitro on parental and CIS-resistant breast cancer cell lines, as well as in vivo using female BALB/c mice implanted with both parental and CIS-resistant breast cancer cells. The triple combination of EA, MET, and CIS effectively inhibited cell division and tumor progression in vivo in both cell lines (Figure 7 and Figure 10), while the EA and MET combination exhibited the highest antiproliferative activity in vitro (Figure 2 and Figure 3). Synergistic interaction between EA and MET was indicated specifically based on CI analysis rather than treatment efficacy comparison alone.

In the MTT assay, both MET and EA inhibited the survival of EMT-6/P cells, which were more sensitive to treatment than their resistant counterparts. In the EMT-6/CPR resistant line, MET demonstrated stronger growth inhibition than EA alone, possibly because EA required a dose higher than 100 µM to reach the IC_50_. However, since a combination treatment was used, higher doses were unnecessary to achieve the inhibitory effect when MET and EA were combined. The combination treatment in the parental breast cancer cell line resulted in a lower IC_50_ value (Figure 2) and a combination index of 0.45, indicating a synergistic interaction between the two treatments (Table 3).

A similar study showed that MET combined with curcumin, a natural product, inhibited cell proliferation and achieved IC_50_ values in EMT-6/P, MCF7, and T47D [28]. Another study reported MET’s ability to overcome cisplatin resistance in triple-negative breast cancer (TNBC) when combined with high-dose resveratrol [38]. Additionally, one study found that EA exhibits antiproliferative and selective cytotoxic properties, inducing apoptosis in Caco-2, MCF-7, Hs 578T, and DU 145 cancer cells, while having no detrimental effect on the survival of healthy human lung fibroblasts [39]. Furthermore, research has demonstrated that EA can effectively mitigate the severe harmful effects of chemotherapy and radiation therapy, and its use may enhance their therapeutic effects when combined with these treatments [40]. Numerous studies support the antiproliferative and tumor-suppressive actions of EA; for example, Cheng et al. concluded that EA suppressed human pancreatic carcinoma PANC-1 cells in both in vivo and in vitro models [41]. Another study showed that EA inhibited cell division, promoted apoptosis, and reduced oxidative stress in gastric cancer AGS cells [42].

A mechanistic understanding is essential to grasp the anti-tumor effects of both agents. Specifically, EA inhibits breast cancer cell growth and exhibits pro-apoptotic activities, including the activation of caspase-9 and caspase-3 in several cancer cell lines, including breast cancer, through multiple proposed molecular mechanisms [11,12,43]. These mechanisms provide a potential biological context for the effects observed in the present study but were not directly investigated here.

One of the key processes involved in maintaining cellular integrity is apoptosis, which ensures that malfunctioning cells do not proliferate and develop into disease. Among the markers of cell death and treatment response in breast cancer, caspase-3 plays a major role in this process; this provides a partial characterization of the cell death mechanism [44]. Therefore, a caspase-3 assay was used to evaluate the effects of EA, MET, and their combination on caspase-3 activation and apoptosis in both parental and CIS-resistant cell lines. The results demonstrated that all treatment groups increased caspase-3 levels, with the highest increase observed in the combination group (EA + MET) in both cell lines (Figure 4 and Figure 5). This finding is consistent with the in vitro CI analysis, which indicated synergistic interactions between EA and MET, with synergy in the parental breast cancer cells and strong synergy in cisplatin-resistant cells (Table 3). In addition, enhanced caspase-3 activation after combination treatment indicates increased apoptotic signaling, which could be one of the underlying cell death mechanisms. However, the enhancement of apoptosis was used as supportive mechanistic evidence rather than as the basis to define synergy. One explanation for the stronger anti-tumor effect observed with the EA + MET combination is that the two compounds may act synergistically on cellular energy regulation and key survival pathways. Metformin is known to activate AMP-activated protein kinase (AMPK) and inhibit mTOR-dependent signaling, which in turn limits anabolic metabolism, cell growth, and survival. Similarly, ellagic acid has also been reported to activate AMPK and reduce mTOR phosphorylation in cancer cells, leading to decreased cell growth and increased apoptosis. Therefore, EA and MET together may provide complementary suppression of pro-survival signaling pathways, which could more effectively inhibit tumor cell proliferation and survival than either agent alone. This overlap may help explain the enhanced antitumor activity observed with the combination in this study [45,46,47]. It should be emphasized that the present study did not directly test these molecular mechanisms. Consequently, their proposed contribution to the observed synergy between EA and MET remains speculative and will require further experimental validation.

In the in vivo study, we introduced a new combination therapy comprising MET, EA, and CIS to compare it with single therapies and to explore the potential sensitizing effect of MET and EA when used together. Treatments were administered via intraperitoneal (i.p.) injection as follows: MET at 80 mg/kg, EA at 45 mg/kg, and CIS at 0.7 mg/kg. The control group received 0.1 mL/mice of MEM media. Results demonstrated that the greatest reduction in tumor size and the highest percentage of mice with no detectable tumors were observed in the triple-therapy group (EA + MET + CIS) and the dual-therapy group (EA + MET), respectively, across both cell lines. Specifically, the percentage change in tumor size was −56.25% and −35.2% in the EMT-6/CPR line, and −68.98% and −73.8% in the EMT-6/P cell line. The noted antitumor effect observed with MET monotherapy in the EMT-6/P model suggests that MET may be a primary contributor to the therapeutic response in vivo. Although the addition of EA and CIS did not significantly enhance tumor volume reduction compared to MET alone, the combination regimens resulted in a higher percentage of mice with no detectable tumors (66.6% vs. 50% for MET alone). In addition, the overall response to the combination regimen may be largely driven by metformin itself, rather than indicating a uniformly additive or synergistic interaction between all three agents. However, the EA + MET + CIS combination showed enhanced antitumor efficacy compared with CIS monotherapy in the EMT-6/CPR model, suggesting a potential for improved treatment efficacy in the cisplatin-resistant setting. However, since no formal interaction analysis was done to assess the triple combination in vivo, these data should be viewed as augmented antitumor efficacy and not proof of pharmacological synergy.

The extra between-group comparisons provide additional evidence for the contribution of each component of the treatment. The improved efficacy of the EA + MET + CIS combination compared to EA and MET monotherapies suggests that the combination of these agents leads to an enhanced antitumor response beyond the activity of the individual compounds. However, the lack of a significant difference between the triple combination and the EA + MET group indicates that the addition of cisplatin did not significantly increase the tumor growth inhibition compared with the dual EA + MET regimen in this model. This result suggests that EA and MET may be the main contributors to the observed anti-tumor activity, and the additional effect of cisplatin may be limited in the current experimental conditions and treatment schedule.

A high percentage of mice with no detectable tumors was noted in the triple-treatment group for EMT-6/CPR (50%), compared to an even higher percentage (66.6%) in the parental breast cancer counterpart for both triple- and dual-therapy groups. This indicates a more potent effect of combination therapy in the parental cell line than in the resistant counterpart. The between-treatment comparisons provided further insight into the relative contributions of each therapeutic component. The triple combination (EA + MET + CIS) was more effective than MET and CIS alone and significantly more effective than the EA + CIS combination, but it was not significantly different from EA alone. These results support the notion that EA may be a significant contributor to the observed antitumor response and that the incremental benefit of combining MET and CIS may depend on the treatment context and specific endpoints. However, a favorable overall response profile was observed for the triple combination, justifying further investigation of this therapeutic strategy.

Although EA demonstrated superior in vitro performance, its efficacy in vivo at reducing tumor size in both cell lines was modest, likely due to the complexity of the tumor microenvironment and possible impact of adaptive resistance mechanisms that may decrease therapeutic efficacy in a physiological setting [48,49].

EA alone exhibited significant antitumor activity; in xenograft models of MCF-7 or MDA-MB-231 cells, oral administration of EA (50 mg/kg) formulated in Tween 80-coated nanoparticles reduced tumor volumes by nearly 60% [26]. Regarding MET, previous studies demonstrated that it enhances cisplatin activity in the triple-negative breast cancer (TNBC) subtype [50].

Studies of MET and EA, as well as their combinations with cisplatin, suggest potential complementary anticancer effects. The interaction between EA and MET was indicated to be synergistic by CI analysis in vitro, while increased caspase-3 activation provided supporting evidence of enhanced apoptotic signaling in this study. For example, the enhanced apoptotic effect can be explained by EA’s ability to induce cell death through caspase-3 activation in ovarian cancer [51], while MET combined with cisplatin also causes significant cleavage (activation) of caspase-3 in human cervical cancer [52]. These findings provide a possible explanation for the increased caspase-3 activation observed in the present study. However, the specific mechanisms underlying these effects were not directly investigated.

We tested the AST, ALT, and Cr levels, which are the biomarkers of liver and kidney function, at the end of the trial, compared to the control. Early indications of organ damage can be found non-invasively with the help of these biomarkers [53]. The triple combination of EA, MET, and CIS led to more significant liver biochemical changes compared to any of the single treatments or double combinations, as evidenced by the high AST and ALT values. The measurement of AST, which was considerably elevated in the group treated with the triple combination as opposed to those treated with EA, MET, and EA + MET, further supports the theory that the use of cisplatin in the combination scheme results in increased levels of stress in the liver during the experiment [54]. As for ALT, it was substantially higher in the triple-combination group than in EA and EA + MET but did not differ significantly compared to MET alone. What is interesting is that the EA + MET combination had no significant effect on any of the liver biochemical parameters when compared to the control group. This could be related to the hepatoprotective properties of EA as evidenced by previous studies [55]. Collectively, significant elevations in ALT and AST were observed in the triple therapy, while creatinine levels remained within normal ranges. The current study did not investigate the mechanism underlying these changes; further evaluation is needed.

A key limitation of this study is that several treatment regimens were not examined in the in vitro assays, most notably the EA + MET + CIS triple combination. Therefore, future research should evaluate the single, double, and triple combinations in cisplatin-resistant cells, employing assays for cell viability, apoptosis, and underlying mechanisms. The positive outcomes observed in the cisplatin-resistant tumor model provide a strong foundation for these follow-up investigations and may help elucidate the mechanisms responsible for the therapeutic effects. Another limitation of the present study is the absence of a cisplatin-alone treatment group in the in vivo cisplatin-sensitive EMT-6/P model. Consequently, the effects of the investigated combination treatments could not be directly compared with cisplatin monotherapy in this tumor model. Future studies should include single cisplatin treatment as well as the relevant individual and combination treatment groups in both cisplatin-sensitive and cisplatin-resistant models. Such comparisons would allow a more comprehensive evaluation of the contribution of each treatment component and further clarify the potential therapeutic advantages of the combination strategies.

Although the combination of EA, MET, and CIS produced a marked antitumor response, the magnitude of its effect did not differ significantly from that observed with EA and MET alone. Consequently, the present results are not statistically sufficient to support the conclusion that adding cisplatin to EA and MET provides an additional antitumor advantage. Nevertheless, the triple regimen achieved pronounced inhibition of tumor growth compared to the control and individual treatment groups, suggesting potential therapeutic relevance. Further studies with larger sample sizes are needed to determine whether cisplatin offers a meaningful incremental benefit.

The selected doses were based on previously tested in vivo regimens to establish a reliable foundation for tolerability and biological activity, while minimizing the risk of severe toxicity or treatment-related mortality. It is important to acknowledge that some studies supporting the selection of drug concentrations were conducted using cancer models other than breast cancer. Therefore, while these studies help justify the concentrations tested in this study, their results may not directly translate to breast cancer. Accordingly, the concentrations used here should be considered exploratory, and further research employing breast cancer-specific models is necessary to validate their translational relevance and to determine clinically relevant exposure levels.

Additionally, further studies should include normal human breast epithelial cells, such as MCF-10A, in addition to breast cancer cell lines to further evaluate the tissue-specific selectivity and safety of the tested treatments. Comparative cytotoxic evaluation in normal and malignant cells would help in establishing the therapeutic selectivity of the treatments and in identifying possible off-target cytotoxic effects.

Although CI analysis indicated synergistic interaction between EA and MET under the tested in vitro conditions, additional studies with expanded dose matrices and mechanistic validation are needed to better characterize the interaction between these agents.

Additional approaches to characterize apoptosis, such as flow cytometry using Annexin V/PI staining and the quantification of other proteins involved in apoptosis, would provide a more comprehensive evaluation of the cell death performed to achieve comprehensive evaluation of the cell death process.

The translational potential of the EA + MET + CIS regimen should be approached with caution. Additional studies incorporating dose–response analyses, pharmacokinetic assessments, extended treatment durations, and comprehensive liver histopathological evaluations are necessary to determine whether the antitumor effects observed can be sustained while minimizing hepatic toxicity. This may provide additional insight into the translational potential of this combination strategy to develop more effective therapies against resistant breast cancer subtypes.

## 5. Conclusions

The results demonstrate that the combination of metformin and EA may enhance the antitumor efficacy of CIS in breast cancer, including CIS-resistant cases. The EA + MET combination demonstrated a synergistic interaction in vitro under the tested conditions, as indicated by Combination Index (CI) analysis. The in vivo results suggest that EA and MET may have potential as complementary agents in a CIS-containing treatment regimen for breast cancer, including CIS-resistant cases. However, the lack of a significant difference between the EA + MET and EA + MET + CIS groups, along with the observed increases in liver enzymes associated with the triple combination, indicates that further research is necessary to more thoroughly evaluate both efficacy and safety before considering clinical use. Further studies are necessary to confirm these results and to elucidate the underlying molecular mechanisms.

## Figures and Tables

**Table 2:** Animal Experiment Treatment Timeline. Each experiment was performed with 66 mice: 30 bearing EMT-6/P and 36 bearing EMT-6/CPR (*n* = 6/group). The treatment groups differed slightly between the two models, as described below.

Group No.	EMT-6/P	EMT-6/CPR	No. Mice	Therapeutic Agent	Treatment Regimen	Volume of Each Dose	Duration
1	√	√	6	EA	45 mg/kg/day (IP inj)	0.1 mL	7 days
2	√	√	6	MET	80 mg/kg/day (IP inj)	0.1 mL	7 days
3	-	√	6	CIS	0.7 mg/kg/day	0.1 ML	7 days
4	√	√	6	EA + MET	45 mg/kg/day EA (IP Inj) & 80 mg/kg/day MET (IP Inj)	+0.1 mL	7 days
5	√	√	6	EA + MET + CIS	45 mg/kg/day EA (IP Inj) & 80 mg/kg/day MET (IP Inj) & 0.7 mg/kg/day Cis	+0.1 mL +0.1 mL	7 days
6	√	√	6	Placebo	-	0.1 mL	7 days

Note: Only the EMT-6/CPR experiment included the cisplatin-only (CIS) treatment group (Group 3); it was not assessed in the EMT-6/P experiment, √ : The corresponding experiment was performed.

**Table 3:** IC_50_ values of EA, MET, and CIS; resistance folds; and combination index (CI) analysis of EA and MET in EMT-6/P and EMT-6/CPR.

In Vitro Model	EA IC_50_ (µM)	MET IC_50_ (µM)	CIS IC_50_ (µM)	EA IC_50_ in Combination with MET (µM)	MET IC_50_ in Combination with EA (µM)	CI	Explanation
EMT6/CPR	100	74.93	9.68	16.31	8.34	0.29	Strong Synergism
EMT6/P	13.74	3.06	3.81	3.57	0.52	0.45	Synergism
Resistant folds	7.278	24.48	2.54	4.56	16.03		
% Reduction in resistance fold	37.3%	34.5%					

**Table 4:** Tumor Size, Percent Change, and Average Weight in EMT-6/CPR Cells Following EA, MET, Combination, and CIS Treatments.

Treatment Group EMT-6/CPR	Av. Initial Tumor Size (mm^3^)	Av. Final Tumor Size (mm^3^)	%Change in Tumor Size (%)	Mice with No Detectable Tumor (%)	Av. Tumor Weight (g)	*p*-Values (EA + MET + CIS vs. Each Treatment)
EA	259.61	267.63	−2.7	33.3	0.195	ns
MET	362.79	469.47	−28.34	16.6	0.298	<0.05
CIS	567.8	467.2	−17.7	16.6	0.43	<0.05
EA + MET	248.11	189.03	−35.2	16.6	0.171	<0.05
EA + MET + CIS	245.98	118.61	−56.25	50	0.088	-
Control	340.89	721.68	109.04	16.6	0.529	<0.01

Abbreviations: Av. = average; mm^3^ = cubic millimeter.

**Table 6:** The effect of EA, MET, and their combinations on serum and kidney biochemical markers in mice. Values are expressed as mean ± SEM. Statistical analysis. *p*-values in the first column set are comparisons with the control, and the second column set are comparisons of the triple-combination group with each treatment group.

Treatment	AST (IU/L)	*p*-Values (Treatment vs. Control)	*p*-Values (EA + MET + CIS vs. Each Treatment)	ALT (IU/L)	*p*-Values (Treatment vs. Control)	*p*-Values (EA + MET + CIS vs. Each Treatment)	Cr (mg/dL)	*p*-Values (Treatment vs. Control)	*p*-Values (EA + MET + CIS vs. Each Treatment)
EA	201.47 ± 1.34	0.001	0.035	34.1 ± 4.2	0.705	0.004	0.15 ± 0.02	0.743	0.779
MET	193.9 ± 3.3	0.005	0.009	45.97 ± 1.4	0.004	0.751	0.158 ± 0.01	0.537	0.962
EA + MET	150.1 ± 10.4	0.956	<0.0001	28.9 ± 4.56	0.450	0.002	0.15 ± 0.02	0.800	0.691
EA + MET + Cis	234 ± 6.08	<0.0001	-	50.3 ± 3.2	0.001	-	0.187 ± 0.001	0.271	-
Control	156.8 ± 7.57	-	<0.0001	29.37± 1.92	-	0.003	0.147 ± 0.01	-	0.691

## Data Availability

The original contributions presented in this study are included in the article. Further inquiries can be directed to the corresponding authors.

## References

[1] Sung H., Filho A.M., Laversanne M., Ferlay J., Siegel R.L., Soerjomataram I., Jemal A., Bray F. (2026). Global cancer statistics 2024: GLOBOCAN estimates of incidence and mortality worldwide for 34 cancers in 186 countries. CA Cancer J. Clin..

[2] Qutob I.A., Soliman A., Aufy H., Elgahamy A.S., Trabelsi R., Shaalan F.T., Omari M., Shehab F., Elgarawany A., Eldaly A.A. (2025). Breast cancer awareness and breast self-examination among females in the Middle East and North Africa: A multinational cross-sectional study. BMJ Oncol..

[3] Freihat O., Sipos D., Kovacs A. (2025). Global burden and projections of breast cancer incidence and mortality to 2050: A comprehensive analysis of GLOBOCAN data. Front. Public Health.

[4] Al Junaidi H.S., Ahmad S.A., Law D., Alshaeri H.K., Talib W.H. (2025). Evaluation of anti-cancer and Immunomodulatory effects of Globe Thistle (Echinops Shakrokii SA Ahmad) extracts: An in vitro and in vivo study. Sci. Rep..

[5] Anand U., Dey A., Chandel A.K.S., Sanyal R., Mishra A., Pandey D.K., De Falco V., Upadhyay A., Kandimalla R., Chaudhary A. (2023). Cancer chemotherapy and beyond: Current status, drug candidates, associated risks and progress in targeted therapeutics. Genes Dis..

[6] Dhar S., Kolishetti N., Lippard S.J., Farokhzad O.C. (2011). Targeted delivery of a cisplatin prodrug for safer and more effective prostate cancer therapy in vivo. Proc. Natl. Acad. Sci. USA.

[7] Li Y., Wang Z., Ajani J.A., Song S. (2021). Drug resistance and Cancer stem cells. Cell Commun. Signal..

[8] Galluzzi L., Senovilla L., Vitale I., Michels J., Martins I., Kepp O., Castedo M., Kroemer G. (2012). Molecular mechanisms of cisplatin resistance. Oncogene.

[9] Petrović N., Matić I.Z., Stanojković T. (2025). Natural compounds and strategies for fighting against drug resistance in cancer: A special focus on phenolic compounds and microRNAs. Am. J. Physiol.-Cell Physiol..

[10] Vattem D., Shetty K. (2005). Biological functionality of ellagic acid: A review. J. Food Biochem..

[11] Čižmáriková M., Michalková R., Mirossay L., Mojžišová G., Zigová M., Bardelčíková A., Mojžiš J. (2023). Ellagic acid and cancer hallmarks: Insights from experimental evidence. Biomolecules.

[12] Golmohammadi M., Zamanian M.Y., Jalal S.M., Noraldeen S.A.M., Ramírez-Coronel A.A., Oudaha K.H., Obaid R.F., Almulla A.F., Bazmandegan G., Kamiab Z. (2023). A comprehensive review on Ellagic acid in breast cancer treatment: From cellular effects to molecular mechanisms of action. Food Sci. Nutr..

[13] Mohammadinejad A., Mohajeri T., Aleyaghoob G., Heidarian F., Kazemi Oskuee R. (2022). Ellagic acid as a potent anticancer drug: A comprehensive review on in vitro, in vivo, in silico, and drug delivery studies. Biotechnol. Appl. Biochem..

[14] Damodaran C., Cho J.-Y., Güngör C. (2025). Therapeutic resistance and combination therapy for cancer: Recent developments and future directions. Sci. Rep..

[15] Pal R.S., Jawaid T., Rahman M., Verma R., Patra P.K., Vijaypal S.V., Pal Y., Upadhyay R. (2025). Metformin’s anticancer odyssey: Revealing multifaceted mechanisms across diverse neoplastic terrains-a critical review. Biochimie.

[16] Saraei P., Asadi I., Kakar M.A., Moradi-Kor N. (2019). The beneficial effects of metformin on cancer prevention and therapy: A comprehensive review of recent advances. Cancer Manag. Res..

[17] Wang L., Zhao X., Fu J., Xu W., Yuan J. (2021). The role of tumour metabolism in cisplatin resistance. Front. Mol. Biosci..

[18] Jawarneh S., Talib W.H. (2022). Combination of ashwagandha water extract and intermittent fasting as a therapy to overcome cisplatin resistance in breast cancer: An in vitro and in vivo study. Front. Nutr..

[19] Jensen J.D., Dunn J.H., Luo Y., Liu W., Fujita M., Dellavalle R.P. (2011). Ellagic acid inhibits melanoma growth in vitro. Dermatol. Rep..

[20] Song C.W., Lee H., Dings R.P., Williams B., Powers J., Santos T.D., Choi B.-H., Park H.J. (2012). Metformin kills and radiosensitizes cancer cells and preferentially kills cancer stem cells. Sci. Rep..

[21] Camacho-Alonso F., Gómez-Albentosa T., Oñate-Sánchez R., Tudela-Mulero M., Sánchez-Siles M., Gómez-García F.J., Guerrero-Sánchez Y. (2020). In vitro study of synergic effect of cisplatin and low molecular weight heparin on oral squamous cell carcinoma. Front. Oncol..

[22] Talib W.H. (2017). Regressions of breast carcinoma syngraft following treatment with piperine in combination with thymoquinone. Sci. Pharm..

[23] Taylor P., Colman L., Bajoon J. (2014). The search for plants with anticancer activity: Pitfalls at the early stages. J. Ethnopharmacol..

[24] Duarte D., Falcão S.I., El Mehdi I., Vilas-Boas M., Vale N. (2022). Honeybee venom synergistically enhances the cytotoxic effect of CNS drugs in HT-29 colon and MCF-7 breast cancer cell lines. Pharmaceutics.

[25] Hamed R.A., Talib W.H. (2024). Targeting cisplatin resistance in breast cancer using a combination of Thymoquinone and Silymarin: An in vitro and in vivo study. Pharmacia.

[26] Kaur H., Ghosh S., Kumar P., Basu B., Nagpal K. (2021). Ellagic acid-loaded, tween 80-coated, chitosan nanoparticles as a promising therapeutic approach against breast cancer: In-vitro and in-vivo study. Life Sci..

[27] Wang N., Wang Z.-Y., Mo S.-L., Loo T.Y., Wang D.-M., Luo H.-B., Yang D.-P., Chen Y.-L., Shen J.-G., Chen J.-P. (2012). Ellagic acid, a phenolic compound, exerts anti-angiogenesis effects via VEGFR-2 signaling pathway in breast cancer. Breast Cancer Res. Treat..

[28] Falah R.R., Talib W.H., Shbailat S.J. (2017). Combination of metformin and curcumin targets breast cancer in mice by angiogenesis inhibition, immune system modulation and induction of p53 independent apoptosis. Ther. Adv. Med. Oncol..

[29] Miller J.P., Egbulefu C., Prior J.L., Zhou M., Achilefu S. (2016). Gradient-based algorithm for determining tumor volumes in small animals using planar fluorescence imaging platform. Tomography.

[30] Talib W.H., AbuKhader M.M. (2013). Combinatorial effects of thymoquinone on the anticancer activity and hepatotoxicity of the prodrug CB 1954. Sci. Pharm..

[31] Ventura G., Bortolotti M., Neveux N., Gusmini X., Nakib S., Sarfati G., Cynober L., De Bandt J.-P. (2017). Influence of an ω3-fatty acid–enriched enteral diet with and without added glutamine on the metabolic response to injury in a rat model of prolonged acute catabolism. Nutrition.

[32] Al Safi M.A., Rashid H.M., Afifi F.U., Talib W.H. (2022). Gaz Alafi: A traditional dessert in the Middle East with anticancer, immunomodulatory, and antimicrobial activities. Front. Nutr..

[33] Waks A.G., Winer E.P. (2019). Breast cancer treatment: A review. JAMA.

[34] Barabas K., Milner R., Lurie D., Adin C. (2008). Cisplatin: A review of toxicities and therapeutic applications. Vet. Comp. Oncol..

[35] Ma S.C., Zhang J.Q., Yan T.H., Miao M.X., Cao Y.M., Cao Y.B., Zhang L.C., Li L. (2023). Novel strategies to reverse chemoresistance in colorectal cancer. Cancer Med..

[36] Jafarzadeh E., Montazeri V., Aliebrahimi S., Sezavar A.H., Ghahremani M.H., Ostad S.N. (2022). Combined regimens of cisplatin and metformin in cancer therapy: A systematic review and meta-analysis. Life Sci..

[37] Engelke L.H., Hamacher A., Proksch P., Kassack M.U. (2016). Ellagic acid and resveratrol prevent the development of cisplatin resistance in the epithelial ovarian cancer cell line A2780. J. Cancer.

[38] Sirhan Z., Abu Nada A., Anabtawi N., Thyagarajan A., Sahu R.P. (2025). Metformin-based combination approaches for triple-negative breast cancer. Pharmaceutics.

[39] Losso J.N., Bansode R.R., Trappey A., Bawadi H.A., Truax R. (2004). In vitro anti-proliferative activities of ellagic acid. J. Nutr. Biochem..

[40] Xue P., Zhang G., Zhang J., Ren L. (2022). Synergism of ellagic acid in combination with radiotherapy and chemotherapy for cancer treatment. Phytomedicine.

[41] Cheng H., Lu C., Tang R., Pan Y., Bao S., Qiu Y., Xie M. (2017). Ellagic acid inhibits the proliferation of human pancreatic carcinoma PANC-1 cells in vitro and in vivo. Oncotarget.

[42] Zheng J., Li C.-F. (2024). Ellagic acid inhibits gastric cancer cells by modulating oxidative stress and inducing apoptosis. Asian Pac. J. Trop. Biomed..

[43] Adams L.S., Phung S., Yee N., Seeram N.P., Li L., Chen S. (2010). Blueberry phytochemicals inhibit growth and metastatic potential of MDA-MB-231 breast cancer cells through modulation of the phosphatidylinositol 3-kinase pathway. Cancer Res..

[44] Dou H., Yu P.Y., Liu Y.Q., Zhu Y., Li F.C., Wang Y.Y., Chen X.Y., Xiao M. (2024). Recent advances in caspase-3, breast cancer, and traditional Chinese medicine: A review. J. Chemother..

[45] Ni X., Shang F.-S., Wang T.-F., Wu D.-J., Chen D.-G., Zhuang B. (2023). Ellagic acid induces apoptosis and autophagy in colon cancer through the AMPK/mTOR pathway. Tissue Cell.

[46] Chomanicova N., Gazova A., Adamickova A., Valaskova S., Kyselovic J. (2021). The Role of AMPK/mTOR Signaling Pathway in Anticancer Activity of Metformin: This paper is dedicated to the 70th anniversary of the founding of Physiologia Bohemoslovaca (currently Physiological Research). Physiol. Res..

[47] Vancura A., Bu P., Bhagwat M., Zeng J., Vancurova I. (2018). Metformin as an anticancer agent. Trends Pharmacol. Sci..

[48] Wu P., Gao W., Su M., Nice E.C., Zhang W., Lin J., Xie N. (2021). Adaptive mechanisms of tumor therapy resistance driven by tumor microenvironment. Front. Cell Dev. Biol..

[49] Qu Y., Dou B., Tan H., Feng Y., Wang N., Wang D. (2019). Tumor microenvironment-driven non-cell-autonomous resistance to antineoplastic treatment. Mol. Cancer.

[50] Lee J.O., Kang M.J., Byun W.S., Kim S.A., Seo I.H., Han J.A., Moon J.W., Kim J.H., Kim S.J., Lee E.J. (2019). Metformin overcomes resistance to cisplatin in triple-negative breast cancer (TNBC) cells by targeting RAD51. Breast Cancer Res..

[51] Chung Y.-C., Lu L.-C., Tsai M.-H., Chen Y.-J., Chen Y.-Y., Yao S.-P., Hsu C.-P. (2013). The inhibitory effect of ellagic acid on cell growth of ovarian carcinoma cells. Evid.-Based Complement. Altern. Med..

[52] Chen Y.-H., Yang S.-F., Yang C.-K., Tsai H.-D., Chen T.-H., Chou M.-C., Hsiao Y.-H. (2021). Metformin induces apoptosis and inhibits migration by activating the AMPK/p53 axis and suppressing PI3K/AKT signaling in human cervical cancer cells. Mol. Med. Rep..

[53] Fingert H., Varterasian M. (2006). Safety biomarkers and the clinical development of oncology therapeutics: Considerations for cardiovascular safety and risk management. AAPS J..

[54] Abd Rashid N., Abd Halim S.A.S., Teoh S.L., Budin S.B., Hussan F., Ridzuan N.R.A., Jalil N.A.A. (2021). The role of natural antioxidants in cisplatin-induced hepatotoxicity. Biomed. Pharmacother..

[55] Aishwarya V., Solaipriya S., Sivaramakrishnan V. (2021). Role of ellagic acid for the prevention and treatment of liver diseases. Phytother. Res..

